# Retinoic Acid Receptors Control Spermatogonia Cell-Fate and Induce Expression of the SALL4A Transcription Factor

**DOI:** 10.1371/journal.pgen.1005501

**Published:** 2015-10-01

**Authors:** Aurore Gely-Pernot, Mathilde Raverdeau, Marius Teletin, Nadège Vernet, Betty Féret, Muriel Klopfenstein, Christine Dennefeld, Irwin Davidson, Gérard Benoit, Manuel Mark, Norbert B. Ghyselinck

**Affiliations:** 1 Institut de Génétique et de Biologie Moléculaire et Cellulaire (IGBMC), Département de Génétique Fonctionnelle et Cancer, Illkirch, France; 2 Centre National de la Recherche Scientifique (CNRS), UMR7104, Illkirch, France; 3 Institut National de la Santé et de la Recherche Médicale (INSERM), U964, Illkirch, France; 4 Université de Strasbourg (UNISTRA), Illkirch Cedex, France; 5 Hôpitaux Universitaires de Strasbourg (HUS), Strasbourg, France; 6 Centre de Génétique et de Physiologie Moléculaire et Cellulaire (GCPhiMC), UMR5534 CNRS, Université de Lyon 1, Villeurbanne, France; Cornell University, UNITED STATES

## Abstract

All-*trans* retinoic acid (ATRA) is instrumental to male germ cell differentiation, but its mechanism of action remains elusive. To address this question, we have analyzed the phenotypes of mice lacking, in spermatogonia, all rexinoid receptors (RXRA, RXRB and RXRG) or all ATRA receptors (RARA, RARB and RARG). We demonstrate that the combined ablation of RXRA and RXRB in spermatogonia recapitulates the set of defects observed both upon ablation of RAR in spermatogonia. We also show that ATRA activates RAR and RXR bound to a conserved regulatory region to increase expression of the SALL4A transcription factor in spermatogonia. Our results reveal that this major pluripotency gene is a target of ATRA signaling and that RAR/RXR heterodimers are the functional units driving its expression in spermatogonia. They add to the mechanisms through which ATRA promote expression of the KIT tyrosine kinase receptor to trigger a critical step in spermatogonia differentiation. Importantly, they indicate also that meiosis eventually occurs in the absence of a RAR/RXR pathway within germ cells and suggest that instructing this process is either ATRA-independent or requires an ATRA signal originating from Sertoli cells.

## Author Summary

Differentiation of spermatozoa from immature germ cells, called spermatogonia, critically depends on retinoic acid (ATRA), the active metabolite of vitamin A that acts though binding to nuclear receptors called RXR and RAR. To understand the mechanism by which ATRA control germ cell differentiation, we generated mice simultaneously lacking all RXR or all RAR specifically in spermatogonia. From their phenotypic analysis, we demonstrate that meiosis does not require a RAR/RXR-dependent pathway in germ cells and propose that this process is either ATRA-independent or requires an ATRA signal originating from somatic cells. We also show that RXR, in the form of dimers with RAR, can drive spermatogonia differentiation through binding to a regulatory region located in the *Sall4* gene. This finding is significant, as the transcription factor encoded by *Sall4* is known to regulate the expression of KIT, a key tyrosine kinase receptor which is frequently deregulated in testicular cancer.

## Introduction

Spermatogenesis is a tightly regulated, cyclical, cell differentiation process, taking place in the seminiferous epithelium of the testis and yielding mature spermatozoa from stem cells. Spermatogonia in the single cell state, known as A single (A_s_) spermatogonia, have traditionally be considered as the main spermatogonia stem cells in the mouse. Upon division, A_s_ spermatogonia give rise either to two new single cells or to a pair of daughter cells called A paired (A_pr_) spermatogonia that do not complete cytokinesis and remain connected through an intercellular bridge. The A_pr_ spermatogonia divide further to form syncytial chains of 4 to 16 A aligned (A_al_) spermatogonia [1]. Collectively, A_s_, A_pr_ and A_al_ (referred to as “undifferentiated spermatogonia”) are present throughout the seminiferous epithelial cycle and retain stem cell properties. Subsequently, A_al_ cells differentiate without mitotic division into A_1_ spermatogonia. Five cell divisions follow A_1_ formation, forming successively A_2_, A_3_ A_4_, In (intermediate) and B spermatogonia. Collectively, A_1_ to B spermatogonia (referred to as “differentiating spermatogonia”) express the prototypic marker KIT [2,3] and differentiate at given stages of the seminiferous epithelium cycle, each step of differentiation being associated with a mitotic division [4].

In rodent, all-*trans* retinoic acid (ATRA), the biologically active form of vitamin A (retinol) is instrumental to spermatogonia differentiation as assessed from vitamin A deficiency studies. In mice fed a vitamin A-deficient (VAD) diet from weaning onwards, all spermatogonia progressively arrest at the A_al_-A_1_ transition, yielding seminiferous tubules that contain only A_al_ spermatogonia and Sertoli cells. Systemic administration of ATRA to VAD mice reinitiates spermatogenesis from mitotically-arrested A_al_ spermatogonia, resulting in their massive differentiation into spermatogonia expressing KIT, the marker of the A_al_-A_1_ transition [2], and resuming their proliferation/differentiation [5,6]. The molecular mechanism through which ATRA controls *Kit* expression is however not yet fully elucidated. Characterizing this mechanism is important not only in the field of reproduction, but also for a better understanding of the biology of testicular germ cell tumors as KIT is also frequently deregulated in seminomas [7].

In cells, ATRA binds to and activates nuclear receptors (RARA, RARB and RARG), which are ligand-dependent transcriptional regulators. They usually function in the form of heterodimers with rexinoid receptors (RXRA, RXRB and RXRG) to control expression of ATRA-target genes through binding to specific sites located in genomic regulatory regions and called retinoic acid response elements (RARE) [8]. In the adult mouse testis, RARG cell-autonomously transduces an ATRA signal required for spermatogonia differentiation. Accordingly, the testes abnormalities observed upon deletion of *Rarg* either in the whole organism or specifically in spermatogonia in sexually mature males are similar to those present in VAD males [9]. As to RXR isotypes, the situation is contrasted. Our *in situ* hybridization (ISH) analyses failed to detect any of them in spermatogonia in the normal mouse testis [10], while another study evidenced RXRA in spermatogonia by immunohistochemistry (IHC) [11]. Thus, either RXR is absent and therefore dispensable for RAR functioning in spermatogonia, as it is the case in Sertoli cells [12], or RXRA is required but its expression level in spermatogonia too low to be detected by ISH.

To discriminate between these two possibilities, we have generated mice lacking all RXR isotypes specifically in spermatogonia from PN5 onwards and analyzed their phenotype. We demonstrate that ablation of all *Rxr* genes arrests differentiation of some spermatogonia at the A_al_-A_1_ transition and recapitulates the full set of defects characteristic of the vitamin A deficiency-induced testis degeneration. We further show that efficient ablation of the 3 *Rar* genes in spermatogonia using the same genetic approach resembles ablation of the 3 *Rxr* genes. Importantly, some A1 spermatogonia still differentiate in these mutants, indicating the existence of a mechanism allowing the A_al_ to A_1_ transition independently of RAR/RXR in germ cells. Along these lines, both meiotic and post-meiotic cells devoid of RAR or RXR are also produced, in contrast to the situation when ATRA synthesis is impaired [13]. We propose that paracrine signals emanating from and transduced in Sertoli cells by ATRA-activated RARA stimulate some A_al_ spermatogonia to become A_1_ and trigger entry into meiosis. We finally provide evidence that RXR and RAR bind to the same *Sall4* regulatory region to control ATRA-dependent expression of SALL4A in the RAR/RXR-dependent spermatogonia. As SALL4A is known to impair ZBTB16-mediated *Kit* repression [14], our study provides novel insights into the molecular mechanism by which ATRA could control KIT expression, and thereby the differentiation of A_al_ into A_1_ spermatogonia *in vivo*.

## Results and Discussion

The ATRA signal is generally transduced by RAR/RXR heterodimers, notably during mouse embryonic development [15]. However, it can also be relayed by RAR independently of RXR, as it is the case in Sertoli cells [12]. Analysis by reverse transcription (RT) coupled to quantitative PCR (qPCR) of mRNA extracted from fluorescent-activated cell sorting (FACS) purified spermatogonia [13] showed that *Rxra* and *Rxrb* mRNA were present in these cells. Thus, contrary to our previous thoughts [10] but in agreement with IHC analyses [11], RXR are actually present in spermatogonia and could well be necessary for their ATRA-induced differentiation. To test for this possibility, we generated mutant mice lacking all three RXR isotypes in undifferentiated spermatogonia and their descendants (hereafter called *Rxra;b;g* ^*Spg–/–*^ mutants) using *Tg(Stra8-cre)* ^*1Reb*^ transgene [16]. In this transgenic line, Cre-mediated ablation occurs as early as post-natal day 3 (PN3), as assessed using a reporter assay (S1 Fig) and in agreement with previous reports [14,16]. The crosses also generated control males devoid of the *Cre* transgene and carrying *lox*P-flanked alleles of *Rxra*, *Rxrb* and *Rxrg*. They did not display testis defects and were hereafter referred to as control mice.

### Loss of RXR in undifferentiated spermatogonia yields age-related testis degeneration

**Fig 1:**
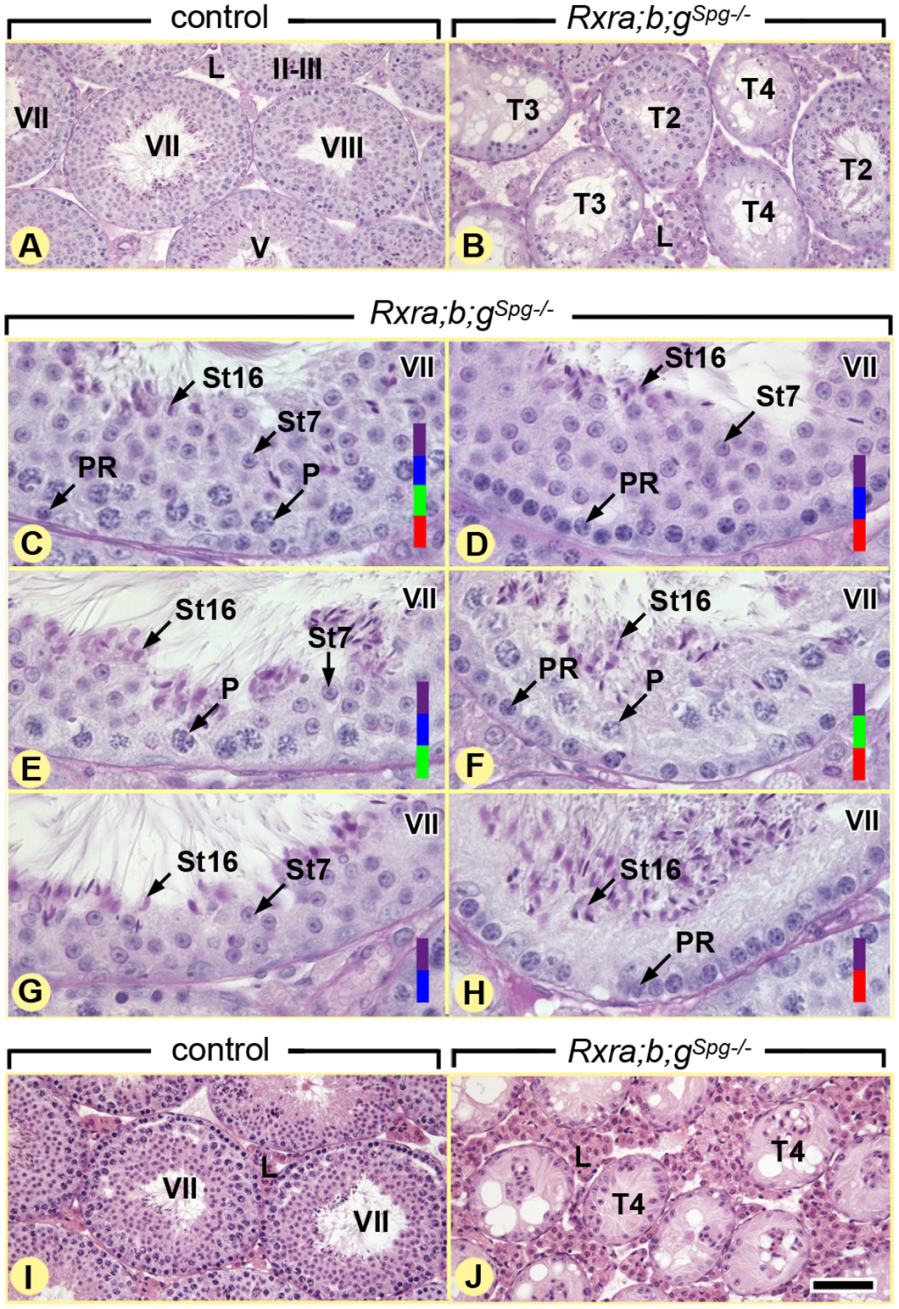
Ablation of RXR in spermatogonia induces age-related testis degeneration. (A,B) Periodic acid-Schiff stains illustrating overviews and (C-H) details of germ cell associations in the seminiferous epithelium of 12 week-old control and *Rxra;b;g* ^*Sgp–/–*^ testes, as indicated. Normal gem cell associations at epithelial stage VII (C) coexist with abnormal associations mimicking, to some extent, this epithelial stage, but lacking: pachytene spermatocytes (D,H), preleptotene spermatocytes (E,G) and round spermatids (F,H). (I,J) Hematoxylin and eosin stain showing overviews of 12 month-old control and *Rxra;b;g* ^*Sgp–/–*^ testes: seminiferous tubules containing only spermatogonia and Sertoli cells represent the end-stage of degeneration in the mutant testes. PR and P, preleptotene and pachytene spermatocytes, respectively; St7 and St16, step 7 and 16 spermatids, respectively; T2, tubule sections lacking generation(s) of germ cells around their entire circumference; T3, tubule sections with disorganization of the germ cell layer; T4, tubule sections containing only spermatogonia and Sertoli cells. Germ cell populations present in a given seminiferous tubule cross-section are highlighted by colored bars: red, preleptotene spermatocytes; green, pachytene spermatocytes; blue, step 7 (round) spermatids; purple, step 16 (elongated, mature) spermatids. Roman numerals indicate the stages of the seminiferous epithelium cycle. Scale bar, 80 μm (A,B and I,J) and 30 μm (C-H).

**Fig 2:**
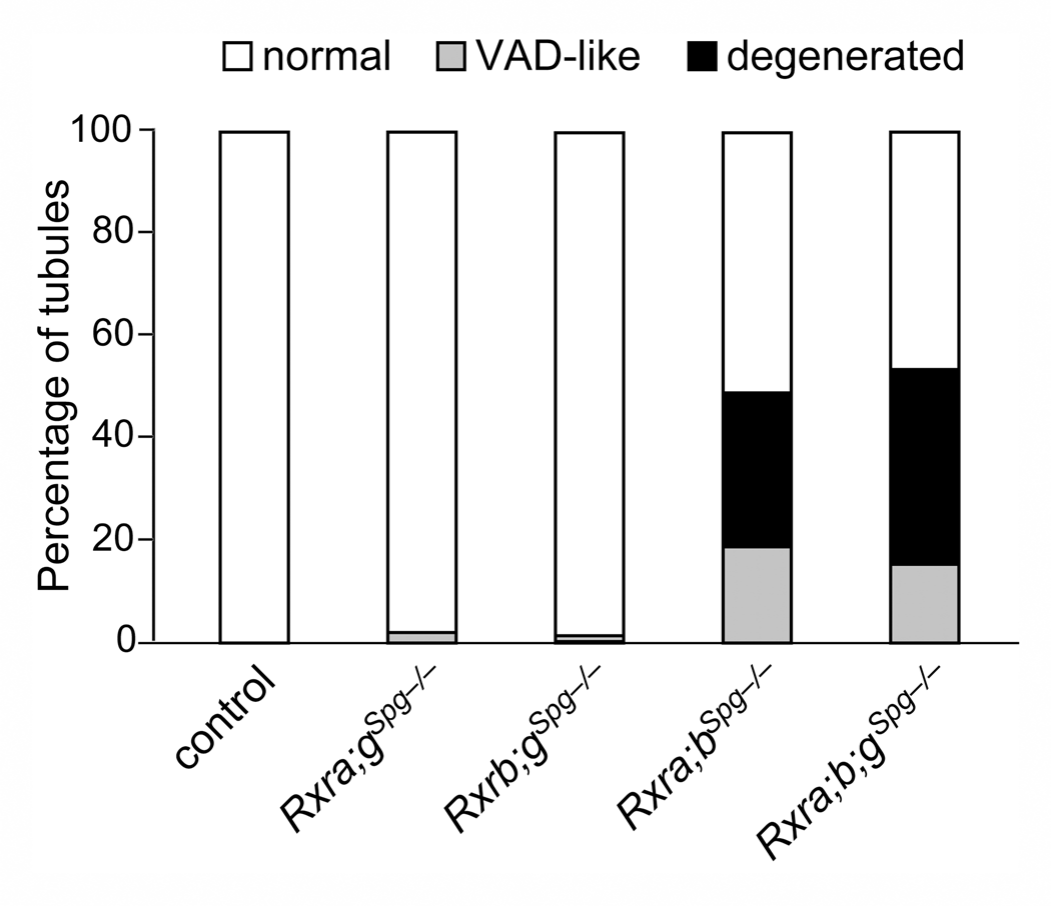
RXRA and RXRB are both instrumental to spermatogonia differentiation. Mean percentages of tubule sections showing normal cellular associations (white bars), abnormal associations resembling the VAD situation with either one or two generations of germ cells lacking (grey bars), and degenerated epithelium containing only spermatogonia and Sertoli cells (black bars) in testes of 12 month-old mice (n = 5) with the indicated genotype. Mice lacking *Rxrg* and either *Rxra* (*Rxra;g* ^*Sgp–/–*^ mutants) or *Rxrb* (*Rxrb;g* ^*Sgp–/–*^ mutants) are marginally affected. In contrast, mice simultaneously lacking *Rxra* and *Rxrb* (*Rxra;b* ^*Sgp–/–*^ mutant) displayed a high proportion of affected tubule sections. Additional ablation of *Rxrg* does not worsen the pathological phenotype (*Rxra;b* ^*Sgp–/–*^ mutant). This indicates that RXRG is dispensable, whereas RXRA and RXRB are both required and exert redundant functions in spermatogonia.

**Fig 3:**
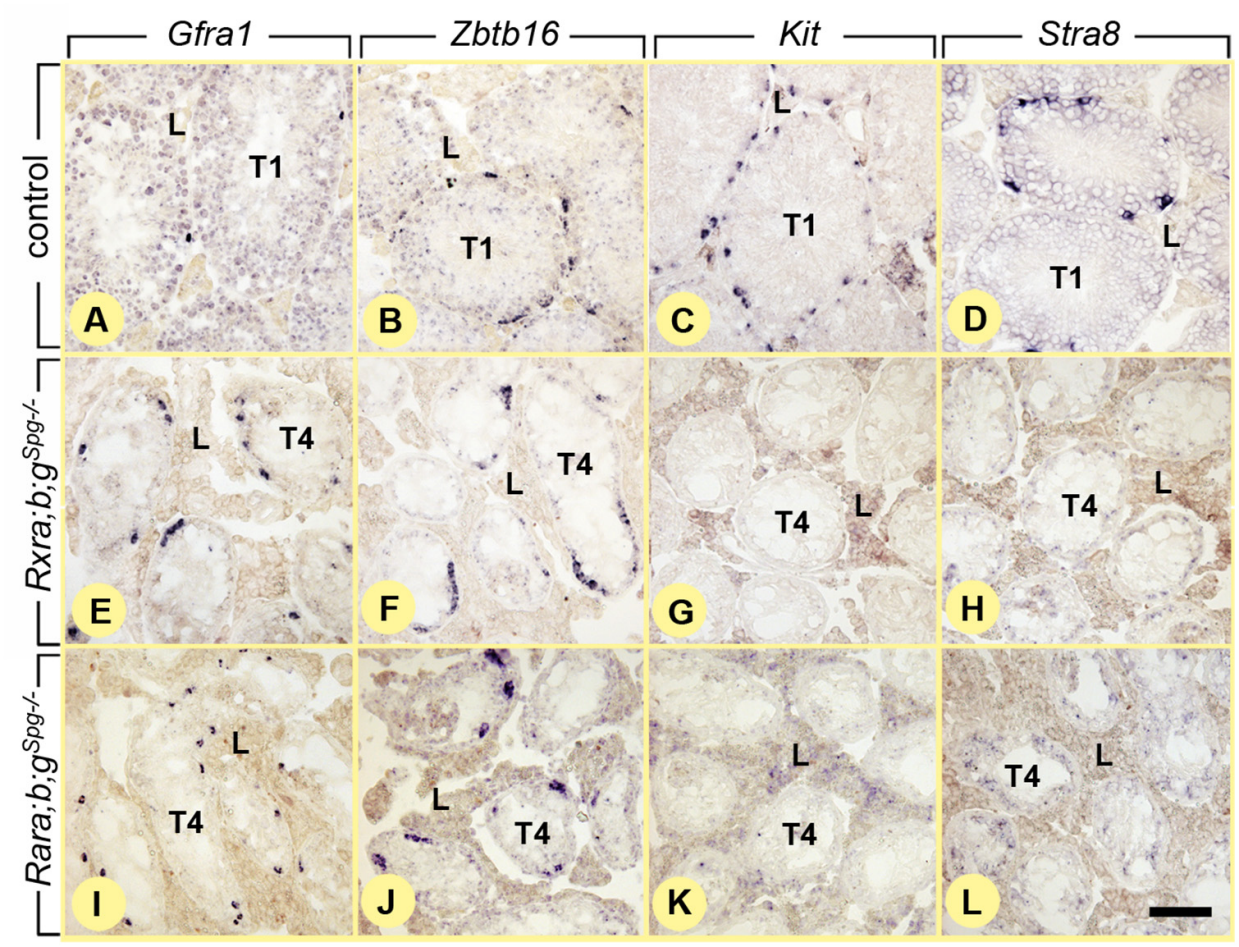
Ablation of RXR blocks spermatogonia are at the undifferentiated stage. *In situ* hybridization with anti-sense probes for *Gfra1*, *Zbtb16*, *Kit* and *Stra8* on histological sections of 12 month-old control (A-D), *Rxra;b;g* ^*Spg–/–*^(E-H) and *Rara;b;g* ^*Spg–/–*^(I-L) testes. Undifferentiated spermatogonia expressing *Gfra1* and *Zbtb16* are found in both control and mutant testes. In contrast differentiating spermatogonia expressing *Kit* and *Stra8* are found in control (C,D) but not in mutant (G,H,K,L) testes. T1, tubule sections showing normal germ cell associations; T4, tubule sections containing only spermatogonia and Sertoli cells; L, Leydig cells. Scale bar: 80 μm.

The different generations of germ cells form cellular associations of fixed composition called epithelial stages. In control testes only the twelve normal epithelial stages (I–XII) [17] were identified (Fig 1A). In contrast, analysis of 12-week-old *Rxra;b;g* ^*Spg–/–*^ mutant testes (n = 5), revealed that, aside from normal epithelial stages (Fig 1C), 36.3 ± 9.6% of the tubule sections exhibited a degenerated seminiferous epithelium (Fig 1B) either lacking a large proportion of germ cells (T3) or containing only spermatogonia and Sertoli cells (T4). In addition, 17.8 ± 3.4% of the tubule sections lacked, around their entire circumference, either one or two generations of germ cells, yielding abnormal variants of the epithelial stages (T2). The missing germ cell layers included: preleptotene spermatocytes (Fig 1E and 1G), pachytene spermatocytes (Fig 1D and 1H), and/or round spermatids (Fig 1F and 1H). Thus, germ cell differentiation appeared altered in *Rxra;b;g* ^*Spg–/–*^ mutants. Analysis of other combinations of compound mutants at the age of 12 weeks revealed that the pathological phenotype was generated solely upon the simultaneous ablation of *Rxra* and *Rxrb* (Fig 2). This indicates that both RXRA and RXRB exert redundant functions in spermatogonia, while RXRG is dispensable. One year-old controls (n = 4) displayed only normal germ cell associations, whereas mutants (n = 4) displayed tubule sections containing only Sertoli cells and spermatogonia (Fig 1I and 1J). The latter expressed molecular markers of undifferentiated spermatogonia such as *Gfra1* and *Zbtb16* [3,18], but not of differentiating spermatogonia such as *Kit* and *Stra8* [2,19] (Fig 3A–3H).

### Ablation of all *Rar* in spermatogonia yields age-related testis degeneration resembling that induced by loss of RXR

The histological defects displayed by *Rxra;b;g* ^*Spg–/–*^ mutants appeared to be much more severe than those observed when *Rarg* and *Rara* genes are deleted using the *Tg(Neurog3-cre)* ^*24Syos*^ transgene [9]. This raised the possibility that RXR isotypes could be instrumental to some aspects of spermatogonia differentiation, independently of RARG and RARA. To test for this hypothesis, we analyzed the outcome of deleting *Rar* genes in spermatogonia by means of the same *Tg(Stra8-cre)* ^*1Reb*^ transgene. Accordingly, mice carrying *lox*P-flanked alleles of *Rara*, *Rarb* and *Rarg* were crossed with *Tg(Stra8-cre)* ^*1Reb*^ mice to generate *Rara;b;g* ^*Spg–/–*^ mutants and their controls.

In 12 week-old *Rara;b;g* ^*Spg–/–*^ mutants (n = 5), 24.5 ± 10.1% of the seminiferous tubule sections were abnormal, amongst which 11.5 ± 5%, identified as variants of the normal epithelial stages, lacked one or two generations of germ cells and 12.9 ± 7.3% exhibited a seminiferous epithelium either with a complete disorganization of the germ cell layers or with spermatogonia and Sertoli cells only (S2 Fig). In one-year-old mutants (n = 3), the seminiferous epithelium consisted only in Sertoli cells and spermatogonia, which expressed genes that are typical of undifferentiated spermatogonia (*i*.*e*., *Gfra1* and *Zbtb16*), but not of differentiating spermatogonia (*i*.*e*., *Kit* and *Stra8*) (Fig 3I–3L). Altogether, these data indicate that age-matched *Rara;b;g* ^*Spg–/–*^ and *Rxra;b;g* ^*Spg–/–*^ mutants display similar, if not identical, phenotypes, including a slow and progressive loss of differentiating germ cells and the presence of spermatogonia blocked at an undifferentiated, A_al_, stage (*i*.*e*., ZBTB16-positive, KIT-negative [2,3]) in aged mutants, both of which are features of the VAD testis [20].

**Fig 4:**
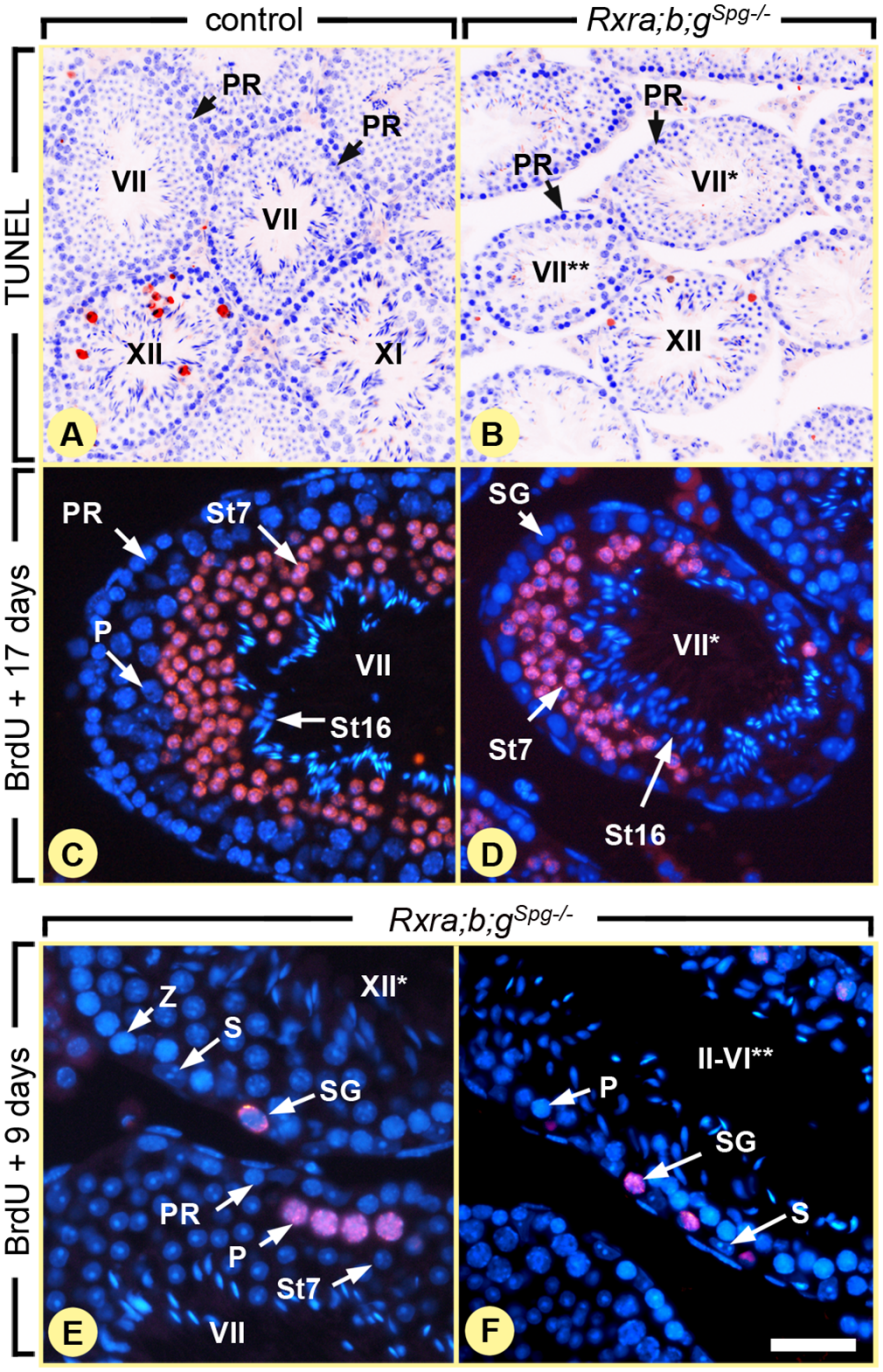
Ablation of RXR in spermatogonia blocks their division, but does not affect meiosis. (A,B) TUNEL assays on histological sections from 8 week-old control and *Rxra;b;g* ^*Spg–/–*^ testis as indicated. Red signals correspond to apoptotic cells and nuclei are counterstained with DAPI (in blue). (C-F) Immunohistochemical detection of BrdU (red signals). After administration, incorporated BrdU has been similarly transferred to spermatids at 17 days (C,D) or to pachytene spermatocytes at 9 days (E,F) both in control and mutant seminiferous tubules. In contrast, spermatogonia retaining BrdU are observed only in mutants (E,F). PR and P, preleptotene and pachytene spermatocytes, respectively; S, Sertoli cells; SG, spermatogonia; St7 and St16, step 7 and 16 spermatids, respectively; Z, zygotene spermatocytes. Roman numerals refer to the stages of the seminiferous epithelium cycle. In mutant testes, one asterisk and two asterisks indicate tubule sections without pachytene spermatocytes and without round spermatids, respectively. Scale bar: 160 μm (A,B), 40 μm (C,D) and 25 μm (E,F).

To further document the similarities between the phenotypes induced by *Rxr* and *Rar* loss-of-functions, we examined the effect of *Rxr* ablation on germ cell apoptosis. Terminal deoxynucleotidyl-transferase dUTP nick end-labeling (TUNEL) assays indicated that apoptosis of preleptotene spermatocytes was not increased in testes of 8 week-old *Rxra;b;g* ^*Spg–/–*^ mutants, relative to age-matched controls (Fig 4A and 4B). Actually, we did not detect a single TUNEL-positive preleptotene spermatocyte in controls and in *Rxra;b;g* ^*Spg–/–*^ mutants (n = 3 males for each genotype; n > 200 preleptotene spermatocytes per testis). Therefore, similarly to the situation in mice lacking *Rara* and *Rarg* in spermatogonia [9], cell-death cannot account for the missing germ cell layers observed in *Rxra;b;g* ^*Spg–/–*^ mutant testes.

We next examined the effect of *Rxr* ablation on the pace of preleptotene spermatocyte differentiation, because any delay or an arrest of this process may lead: (i) to the disappearance of pachytene spermatocytes through their normal differentiation into round, step 7, spermatids after one cycle of the seminiferous epithelium (*i*.*e*., 8.6 days), then (ii) to the disappearance of step 7 spermatids through their normal transformation into mature, step 16, spermatids after completion of a second cycle. Thus, we evaluated the duration of meiotic phase of spermatogenesis after 5-bromo-2'-deoxyuridine (BrdU) incorporation into S-phase nuclei. In adult testis, BrdU is mainly incorporated into B spermatogonia and preleptotene spermatocytes [20]. We fate-mapped the BrdU-labeled descendants of these cells 9 and 17 days after injection of the tracer. At the latter time-point, the most advanced, BrdU-positive, cell-type was step 7 spermatids in both control and *Rxra;b;g* ^*Spg–/–*^ testes (n = 3 for each genotype; 8 week-old) and there was no retained labeling in any spermatocyte (Fig 4C and 4D). Thus, similarly to the situation in mice lacking *Rara* and *Rarg* in spermatogonia [9], the duration of meiosis is not altered in *Rxra;b;g* ^*Spg–/–*^ mutant testes. In this context, it seems logical to assume that ablation of *Rxr* genes induces germ cell depletion solely through altering the spermatogonia proliferation/differentiation process. Accordingly, BrdU was detected in *Rxra;b;g* ^*Spg–/–*^ testes, 9 days after its incorporation, in cells displaying histological features of spermatogonia (Fig 4E and 4F). BrdU was never detected in spermatogonia of control testes at this time-point after injection because its amount becomes diminished by half in each daughter cell upon cell-division, yielding a progressive decrease of the signal over time and its absence 9 days after BrdU incorporation. This observation indicates that some of the spermatogonia that had incorporated BrdU in the *Rxra;b;g* ^*Spg–/–*^ testes did not divide further or divided more slowly than in control testes, as it is the case for spermatogonia lacking *Rar* [9] and in VAD testis [20]. Our study shows therefore that the outcomes of ablating RAR or RXR in spermatogonia are identical at the histological level.

### Differentiation of A_1_ spermatogonia and meiosis still can occur when RAR and RXR are absent in germ cells

Given the central role assigned to ATRA in spermatogonia differentiation and in male meiosis [13,21], it was surprising that differentiation of only some spermatogonia was impaired, and that meiosis always proceeded normally in the absence of either *Rar* or *Rxr*. To exclude the possibility that Cre-mediated excision was mosaic, thereby resulting in the absence of RAR or RXR in some, but not all, differentiating germ cells, we analyzed their expression in *Rara;b;g* ^*Spg–/–*^ and *Rxra;b;g* ^*Spg–/–*^ testes. RT-qPCR analysis of whole testis RNA showed that the amount of *Rarg* transcripts was markedly reduced (4-fold) in *Rara;b;g* ^*Spg–/–*^ mutants, as early as PN5 (S3 Fig). Consistent with this finding, IHC analyses indicated a total absence of RARG in germ cells of *Rara;b;g* ^*Spg–/–*^ mutants, at PN5 and at PN60 (S3 Fig). In fact 100% of spermatogonia and of preleptotene spermatocytes were devoid of RARG in *Rara;b;g* ^*Spg–/–*^ mutants. Efficient loss of RARA and RARB in these mutants can be assessed neither by RT-qPCR because they are not, or only weakly, expressed in spermatogonia nor by IHC analyses because reliable antibodies are not available [9,10]. However, efficient ablation of *lox*P-flanked *Rara* and *Rarb* alleles was assessed at the genomic level, using FACS-purified germ cell populations [22]. PCR analysis demonstrated that excised (null, L–), but not conditional (L2) alleles, were detected in genomic DNA isolated from spermatogonia, spermatocytes and spermatids of PN60 mice bearing the *Tg(Stra8-cre)* ^*1Reb*^ transgene (S3 Fig). Together, these data indicate that ablation of all 3 *Rar* genes was efficient in all germ cells, as early as PN5. As the outcome of ablating all RAR in spermatogonia appears very close to that induced upon *Rarg* knockout (*Rarg* ^*–/–*^ mutants) [9], our data suggest that RARG is the major functional RAR isotype in spermatogonia. Similar results were obtained in *Rxra;b;g* ^*Spg–/–*^ mutants, with *Rxra* transcript amounts markedly reduced (4- to 5-fold) as early as PN5 (S3 Fig).

**Fig 5:**
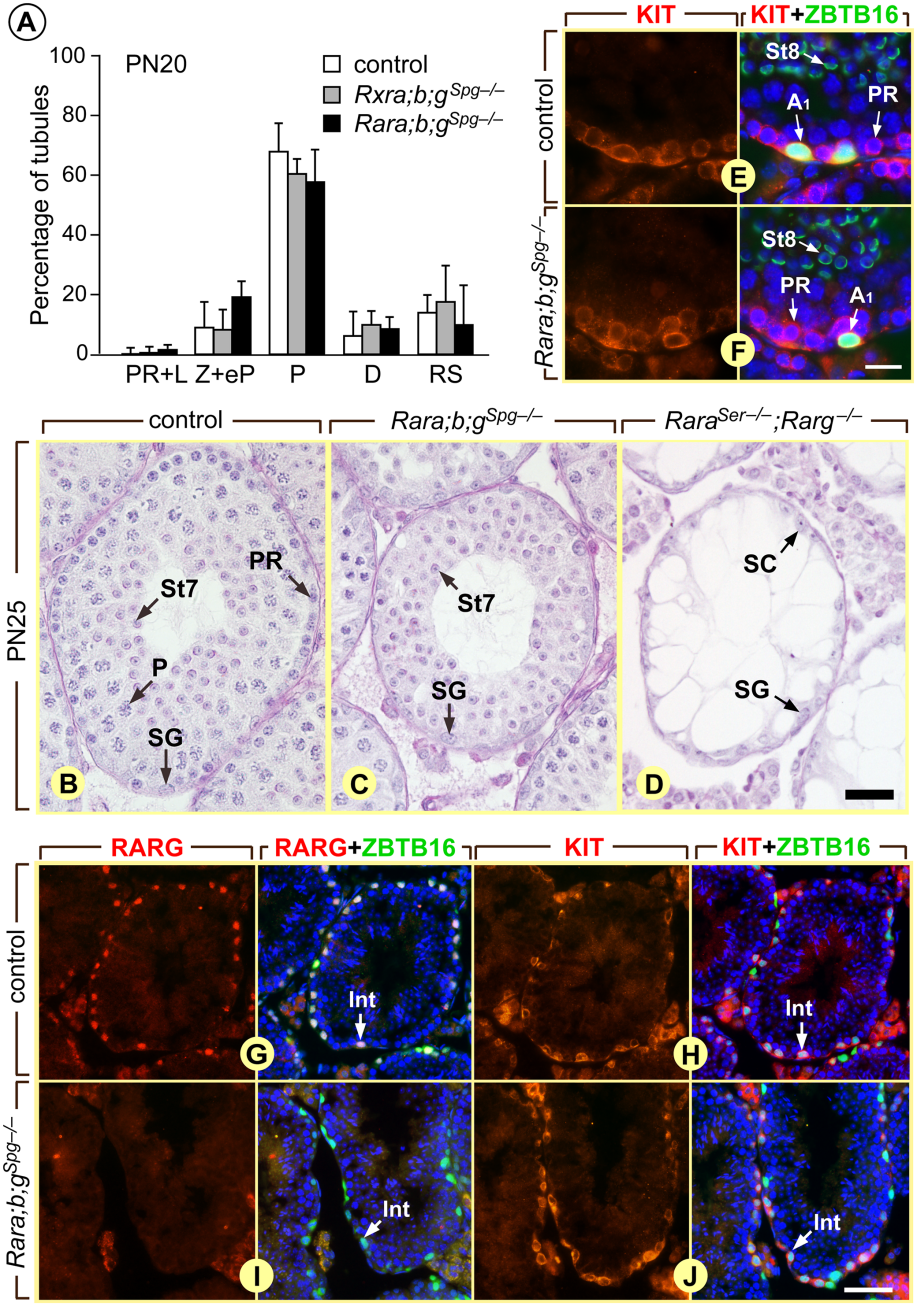
Ablation of either RXR or RAR in spermatogonia does not alter the first round of spermatogenesis. (A) Percentages of seminiferous tubule cross-sections in which preleptotene/leptotene (PR+L), zygotene and early pachytene (Z+eP), late pachytene (P) and diplotene (D) spermatocytes or round spermatids (RS) represent the most advanced germ cell-types in control (white bars), *Rxra/b/g* ^*Spg–/–*^(black bars) and *Rara/b/g* ^*Spg–/–*^(grey bars) testes at post-natal day 20 (PN20). The bars represent mean ± s.e.m. (n = 4–5). (B-D) Histological sections of seminiferous from post-natal day 25 (PN25) control, *Rara/b/g* ^*Spg–/–*^ and *Rara* ^*Ser–/–*^/*Rarg* ^*–/–*^ mice stained with hematoxylin and eosin. Note the absence of spermatocytes (C) or of all meiotic and post meiotic germ cells (D) in the mutant testes. (E-J) Detection of spermatogonia expressing KIT (red signal in E, F, H and J) and RARG (red signal in G and I) in control and *Rara/b/g* ^*Spg–/–*^ testes at 6 weeks of age. ZBTB16 (green nuclear signal in E, F, H and J) identifies spermatogonia. Alexa Fluor 488-conjugated peanut agglutinin (in E and F) labels the acrosomal system of spermatids, allowing precise identification of the stage of the seminiferous epithelium cycle. (G and H) and (I and J) represent consecutive histological sections. All sections were counterstained with 4′,6-diamidino-2-phenylindole (DAPI) to label nuclei (blue signal). A_1_, A_1_ spermatogonia, based on their presence in seminiferous tubule sections that contain both preleptotene spermatocytes (KIT-positive and ZBTB16-negative) and step8 spermatids. Int, intermediate spermatogonia, based on cell density co-expression of KIT and ZBTB16, and peanut hemagglutinin staining of acrosomes on consecutive sections. PR, P, preleptotene and pachytene spermatocytes, respectively; SG, spermatogonia, St7 and St8, step 7 and step8 round spermatids. Scale bars: 30 μm (B-D), 20 μm (E and F) and 55 μm (G-J).

Assuming that the excision of *Rar* and *Rxr* genes was complete from PN5 onwards, the impact of RAR or RXR loss-of-functions during the pubertal development of the testis was evaluated at PN20, *i*.*e*., when the first post-meiotic cells appear. At this developmental stage, control, *Rxra;b;g* ^*Spg–/–*^ and *Rara;b;g* ^*Spg–/–*^ mutant testes (n = 4 for each genotype) were indistinguishable: in both situations, late pachytene and diplotene spermatocytes represented the most advanced germ cell in the vast majority of tubule sections (Fig 5A). These results indicate that the spermatocytes present at PN20 in the mutants testes, appeared in due time, likely because the spermatogonia from which they derived started to differentiate before PN3, at a time when *Rar* or *Rxr* genes were not yet knocked out. More importantly, they also indicate that all preleptotene spermatocytes initiated meiosis normally (around PN8), at a time when they were devoid of RAR or RXR since ablation was obvious from PN5 in their precursors (see above). Analyzing the seminiferous epithelium later during pubertal development revealed the occurrence of abnormal cellular associations at PN25: few tubule sections in mutant testes displayed spermatogonia associated with round spermatids but without the intervening layers of preleptotene and pachytene spermatocytes (Fig 5B and 5C). This observation confirms the initial wave of A_1_ spermatogonia differentiation was not affected (yielding step 7 spermatids at PN25), and suggests the second wave was arrested (or delayed) in few tubules at some point before meiosis, leading to the absence of spermatocytes at PN25. However, the presence of normal cellular associations in the majority of tubule sections indicates that both A_1_ spermatogonia differentiation and meiosis occurred, despite absence of RAR or RXR in germ cells (see above). In keeping with this, KIT-positive A_1_ spermatogonia were found at stages VII-VIII of the seminiferous epithelium cycle in *Rara;b;g* ^*Spg–/–*^ mutants at PN60, similarly to the situation in control mice (Fig 5E and 5F).

The reason why ablation of RAR or RXR in germ cells affects only a fraction of A_1_ spermatogonia is unclear. In some cases the A_1_ transition appears to take place in due time, yielding normal cell associations, while in other instances no A_1_ are formed and A_al_ spermatogonia have to wait one (or several) epithelial cycles to become A_1_, yielding seminiferous tubule segments with missing generations of spermatocytes and spermatids. Moreover, spermatogonia do not transition at random because, as a result of stage-dependent cell divisions [4], extensive rows of differentiating, KIT-positive, RARG-negative, spermatogonia were observed at stages I-VI of the seminiferous epithelium cycle in *Rara;b;g* ^*Spg–/–*^ mutants, as it is the case in control males (Fig 5G–5I). An unknown signal, distinct from ATRA, may operate as a backup only in the context of *Rara;b;g* ^*Spg–/–*^ and *Rxra;b;g* ^*Spg–/–*^ mutants to promote spermatogonia differentiation. Alternatively, two populations of A_al_ spermatogonia may normally exist in the seminiferous epithelium at stages VII-VIII, one requiring RAR/RXR to transition, the other one being committed to become A_1_, independently of RAR/RXR heterodimers. As A_1_ spermatogonia were less and less often generated with aging and no longer observed in old *Rara;b;g* ^*Spg–/–*^ and *Rxra;b;g* ^*Spg–/–*^ mutants, the second population may become depleted with time resulting in a complete arrest of spermatogenesis. Given the pivotal role of ATRA and the precise timing in spermatogonia differentiation at stage VII-VIII of the seminiferous epithelium cycle [4], instructing the transition in this second population may also require ATRA. We propose that an ATRA signal transduced by RARA in Sertoli cells stimulates some A_al_ spermatogonia to become A_1_ even though no RAR/RXR pathway is functional within these latter cells. In keeping with this proposal, it worth noting that the seminiferous epithelium of mice lacking both RARA in Sertoli cells and RARG in spermatogonia (*Rara* ^*Ser–/–*^;*Rarg* ^*–/–*^ mutants) was found to consist only in Sertoli cells and undifferentiated spermatogonia (Fig 5D), as it is the case when ATRA synthesis is specifically abolished in Sertoli cells [13].

More surprisingly, following the A_al_ to A_1_ transition, germ cell differentiation progressed at a normal pace despite the lack of RAR or RXR inside these cells. This indicates that initiation (and progression) of meiosis can proceed even in the absence of a functional ATRA signaling pathway in spermatocytes. If one considers that ATRA is as a mandatory meiosis-inducing substance in vertebrates [23], then our finding necessarily implies that the ATRA-dependent pathway instructing preleptotene spermatocytes is not autocrine in nature, as previously proposed [13], but instead operates in Sertoli cells. In this context, ATRA would control the synthesis by Sertoli cells of yet unknown, intermediate, secreted factor(s) acting on spermatocytes to trigger meiosis. Alternatively, the possibility exists that meiotic initiation does not require ATRA in male germ cells, as it was shown to be the case in female germ cells [24]. Discriminating between these two possibilities awaits further investigations. Regardless of the scenario, our findings indicate that expression of *Stra8* does not require a RAR/RXR-dependent signaling pathway in preleptotene spermatocytes, even though this receptor heterodimer can efficiently bind to RARE in the *Stra8* promoter [13,24].

### RARG/RXR heterodimers bind to *Sall4* and induce expression of SALL4A in spermatogonia

The similarities between *Rar* and *Rxr* gene ablations indicate that RAR and RXR exert convergent functions in spermatogonia, and support the possibility that ATRA signaling in these cells involves RAR/RXR heterodimers. They also indicate that RXR in spermatogonia are unlikely to play a role other than controlling differentiation in conjunction with RAR. To test whether RAR and RXR were actually recruited to an endogenous gene promoter in spermatogonia *in vivo*, we performed immunoprecipitation (IP) of these nuclear receptors using chromatin from PN5 wild-type testes, followed by qPCR analysis of the recovered DNA fragments, and assessed binding to *Stra8*, which is proposed as a RAR target-gene in spermatogonia [21]. Both anti-RAR and anti-RXR antibodies were able to precipitate, with similar efficiencies, the DNA sequences containing the RAR binding sites of the *Stra8* promoter (S4 Fig). These data further support the notion that RAR/RXR heterodimers can be the functional units transducing the ATRA-signal in spermatogonia. However, although STRA8 promotes spermatogonia differentiation, it is not strictly required for this process [19,25,26] and its expression does not appears to be dependent upon RAR/RXR-signaling (see above). Thus, effectors acting downstream of ATRA and distinct from STRA8 likely account for the A_al_ to A_1_ spermatogonia transition.

To gain insights into the genetic cascade controlled by RAR/RXR heterodimers and aside from STRA8, we set up an experiment aimed at identifying ATRA-controlled genes in spermatogonia. To this purpose, we used *Aldh1a1-3* ^*Ser−/−*^ mutants as a model, in which all retinaldehyde dehydrogenase activity is ablated in Sertoli cells. These mutants were chosen because (i) their spermatogonia differentiation is blocked at the A_al_ stage, (ii) A_al_ spermatogonia express RARG [9] and (iii) A_al_ spermatogonia are poised to differentiate into A_1_ spermatogonia upon activation of ATRA signaling [13]. We treated organotypic cultures of *Aldh1a1-3* ^*Ser−/−*^ testes with the RARG-selective agonist BMS961 (n = 5) or with its vehicle (n = 5) for 6 hours and extracted mRNA. Microarray expression profiling identified only a few transcripts that were differentially expressed upon activation of RARG, amongst which *Sall4*. This gene encodes two isoforms named SALL4A and SALL4B [27]. They are zinc-finger transcription factors, which participate in regulatory networks and are critical for cell fate decisions and lineage specification [28,29]. In the mouse testis, their expression is restricted to spermatogonia [30,31] and mice deficient for *Sall4* in these cell-type display testis defects that resemble those observed in *Rxra;b;g* ^*Spg–/–*^ and *Rara;b;g* ^*Spg–/–*^ mutants, namely loss of differentiating, KIT-positive, spermatogonia and of meiotic cells [14]. Thus *Sall4* gene appears particularly relevant to ATRA-induced spermatogonia differentiation.

**Fig 6:**
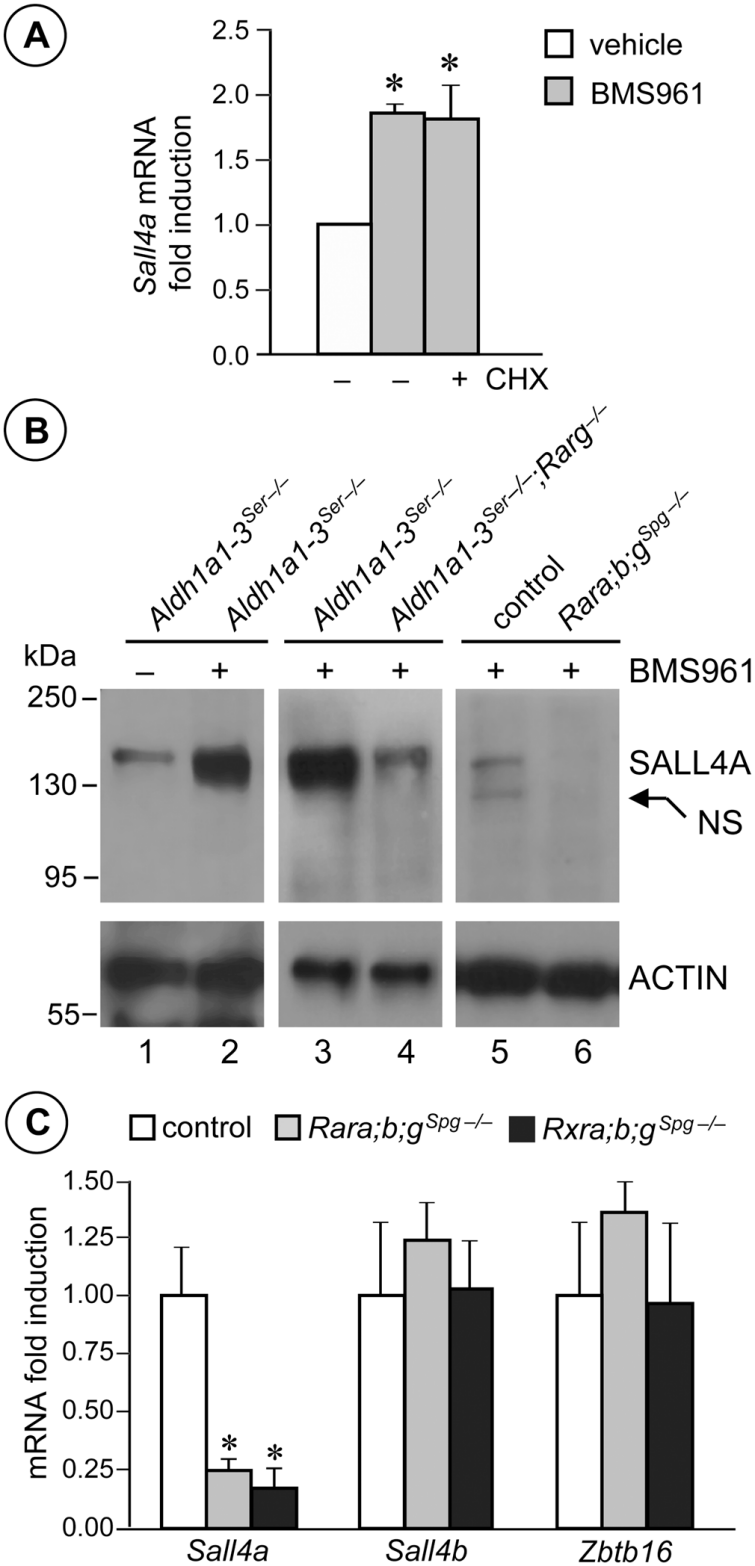
*Sall4a* expression in undifferentiated spermatogonia is controlled by ligand-activated RARG. (A) Relative expression of *Sall4a* mRNA quantified by RT-qPCR in *Aldh1a1-3* ^*Ser−/−*^ testes cultured in the absence (−) or in the presence (+) of cycloheximide (CHX) and treated for 6 hours with vehicle (white bar) and BM961 (grey bars). Error bars represent s.e.m. (n = 5); * *p* < 0.05. (B) Western blot analysis of protein extracts from testes of mutants as indicated treated with BMS961 (+) or with vehicle (−), using anti-SALL4 or anti-ACTIN antibodies. NS points to an unspecific signal. (C) Relative expression of *Sall4a*, *Sall4b* and *Zbtb16* mRNA quantified by RT-qPCR in whole testes from control (white bars), *Rara;b;g* ^*Spg−/−*^ (grey bars) and *Rxra;b;g* ^*Spg−/−*^ (black bars) mice at PN60. Error bars represent s.e.m. (n = 5); * *p* < 0.05.

We confirmed by RT-qPCR that *Sall4a* mRNA steady state level was increased upon BMS961 administration in *Aldh1a1-3* ^*Ser−/−*^ testes, without the need for intermediate protein synthesis as this increase occurred in the presence of cycloheximide (Fig 6A). Western-blot analysis of protein extracts from *Aldh1a1-3* ^*Ser−/−*^ testes revealed that SALL4A protein level was increased by BMS961-activated RARG (Fig 6B, compare lane 1 to 2); this increase was prevented in mice additionally carrying a *Rarg* knock-out (Fig 6B, compare lane 3 to 4) and was not observed in BMS961-treated *Rara;b;g* ^*Spg–/–*^ mutants (Fig 6B, compare lane 5 to 6). In addition, *Sall4a* mRNA levels were significantly decreased in whole testis of *Rara;b;g* ^*Spg–/–*^ and *Rxra;b;g* ^*Spg–/–*^ mutants at PN60, while *Sall4b* and *Zbtb16* mRNA levels were unchanged (Fig 6C). The finding that *Sall4b* mRNA level was not altered is in keeping with previous reports showing that SALL4B is expressed at a constant level in spermatogonia [31,32]. Altogether our results indicate that (i) *Sall4a* expression is decreased in testes of mice lacking RAR or RXR in spermatogonia (*Rara;b;g* ^*Spg–/–*^ and *Rxra;b;g* ^*Spg–/–*^ testes); (ii) SALL4A is detected at a low level in the seminiferous epithelium of mice deficient in ATRA (*Aldh1a1-3* ^*Ser−/−*^ testes), but at a high level when RARG is activated by BMS961 in these mice (*Aldh1a1-3* ^*Ser−/−*^ testes treated with BMS961), except when RARG is lacking (*Aldh1a1-3* ^*Ser−/−*^;*Rarg* ^*−/−*^ testes, treated with BMS961); and (iii) SALL4A is not detected in testes of adult mice lacking RAR in spermatogonia even in the presence of the RARG agonist (*Rara;b;g* ^*Spg–/–*^ testes, treated with BMS961). Altogether, these data indicate that *Sall4a* expression is controlled by ATRA-activated RARG in spermatogonia.

**Fig 7:**
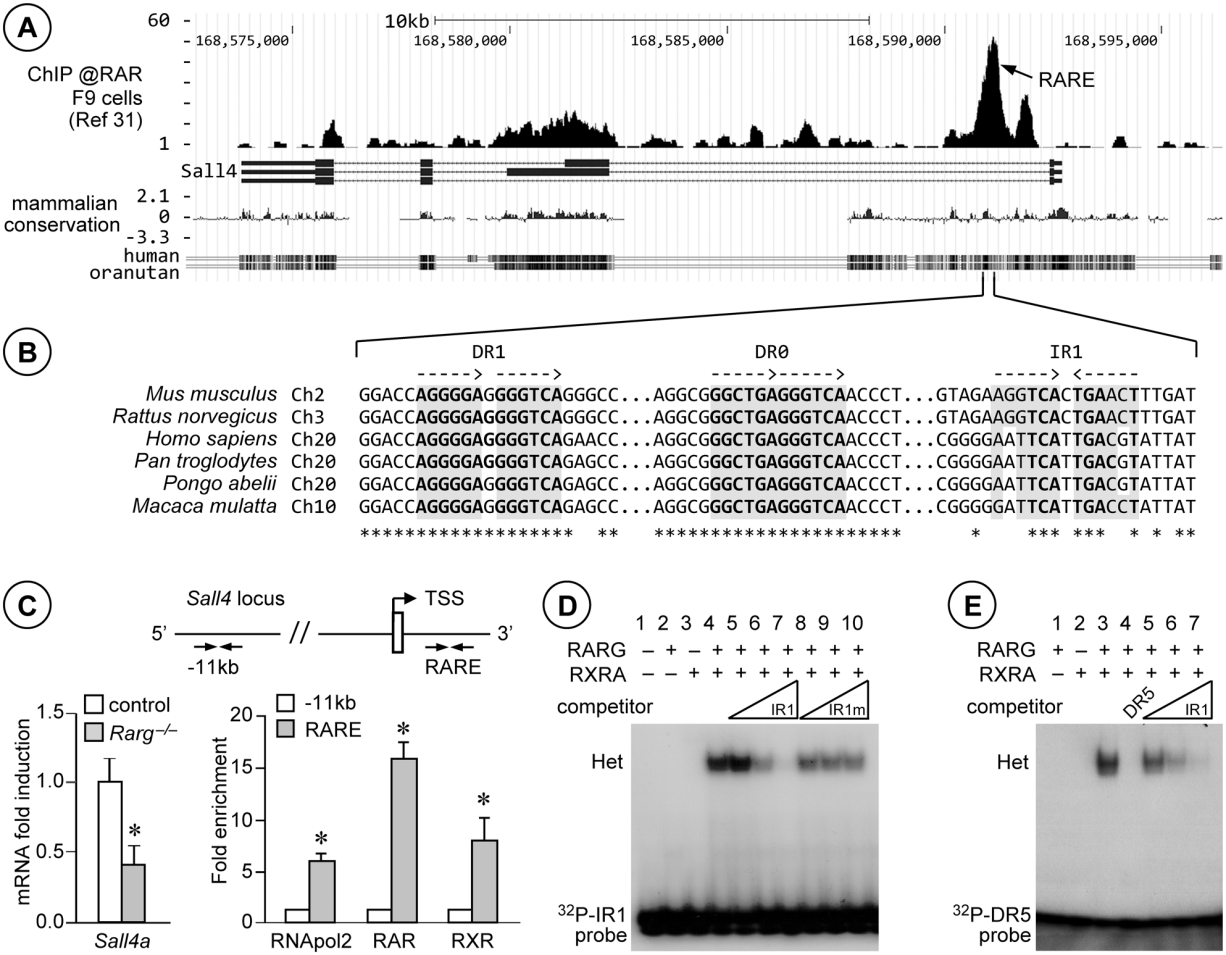
RARG/RXRA heterodimers bind to *Sall4* in testis chromatin, on an IR1 motif located in the first intron. (A) UCSC Genome Browser snapshot of the *Sall4* locus in NCBI37/mm9 assembly, including tracks for anti-RAR ChIP-seq [34], RefSeq genes and mammalian conservation (from top to bottom). RARE points to the RAR-binding region identified according to Moutier et al. [34]. (B) Alignments of the DNA sequences from the indicated species and corresponding to the region containing the RAR-binding region in mouse *Sall4*. The dotted arrows indicate orientations of the core motifs. Stars and grey boxes highlight the DNA residues and the RAR binding sequences that are conserved in all 6 species, respectively. (C) Left panel: relative expression of *Sall4a* mRNA quantified by RT-qPCR in whole testes from control (white bars) and *Rarg* ^*−/−*^ (grey bars) mice at PN5. Error bars represent s.e.m. (n = 9); * *p* < 0.05. Right panel: schematic representation of *Sall4* locus and analysis of DNA recovered from testis chromatin immunoprecipitated using antibodies directed against RNApol2, all RAR or all RXR isotypes (RAR or RXR, respectively). The untranslated exon and the transcription start site (TSS) are depicted by an open box and a broken arrow, respectively. The locations of primers used are indicated at −11 kb and in the RAR-binding region (RARE). Mean fold enrichment of three experiments at RARE binding site (grey bars) is relative to the amount of DNA recovered at −11 kb (set at 1, white bars). Error bars represent s.e.m. (n = 4 to 5); * *p* < 0.05. (D) EMSA showing that RARG/RXRA heterodimers (Het) bind to the ^32^P-labelled IR1 of *Sall4* (lane 4). Binding is competed when increasing amounts of unlabeled IR1 are added to reaction (lanes 5–7), but not when a mutated form IR1 is added (IR1m, lanes 8–10). ^32^P-IR1 probe indicates unbound DNA. (E) EMSA showing that RARG/RXRA heterodimers (Het) bound to the DR5 of *Rarb* (lane 3) are competed both when unlabeled DR5 (lane 4) or increasing amounts of IR1 (lanes 5–7) are added to reaction. ^32^P-DR5 probe indicates unbound DNA.

Using data sets locating RAR-occupied sites genome-wide in several cell-types [33,34], we identified a 700 bp-long RAR-binding region located in the first intron of *Sall4* (RARE, Fig 7A). This DNA fragment contained a RAR binding sequence called IR1, consisting of inverted repeats (two core motifs 5’-RGKTSA-3’ oriented head-to-tail) separated by 1 bp (Fig 7B), as well as two additional sites called DR1 and DR0 (direct repeats of the core motif separated by 1 and 0 bp, respectively). We performed triplicate IP experiments with anti-RAR and anti-RXR antibodies using chromatin extracted from PN5 wild-type mouse testes as substrate. At this developmental stage, *Sall4a* expression was dependent upon RARG (Fig 7C, left panel). We analyzed the immuno-precipitated chromatin fragments by qPCR and evidenced robust binding of both RAR and RXR *in vivo*, in a 106bp-long region restricted to chr2:168,591,142–168,591,247 (NCBI37/mm9) in *Sall4* (RARE, Fig 7C, right panel). To further confirm interaction of IR1 with RAR/RXR heterodimers, we performed electrophoretic mobility shift assays (EMSA) (Fig 7D). They revealed that RARG isotype in combination with RXRA isotype (lane 4), but neither RARG nor RXRA alone (lanes 2 and 3, respectively), bound the radiolabelled IR1 sequence. Binding was competed by increasing amounts of unlabeled IR1 (lanes 5–7), but not by IR1m bearing point-mutations in the first core motif (lanes 8–10). They also showed that unlabeled IR1 efficiently competed binding of RARG/RXRA heterodimers to the radiolabeled, canonical, RAR binding site of *Rarb* gene (called DR5, Fig 7E). The data suggested therefore that RARG/RXRA heterodimers could enhance expression of SALL4A through binding to an IR1 motif located in *Sall4* intron. This motif appeared moderately well-conserved in mouse, rat, human and primate genomes (Fig 7B), but single mismatches do not necessarily abrogate RAR/RXR binding, even when located at highly conserved positions [33]. The DR1 and DR0 were also able to bind RARG/RXRA heterodimers (S5 Fig). Their sequences were even well-conserved across the species than that of IR1 (Fig 7B). Thus the RAR binding region in *Sall4* belongs to the category of “composite elements”, the functionality of which has already been demonstrated [34]. Interestingly, SALL4A is also expressed in human spermatogonia [35]. Moreover, Fertilysin (N,N’-1,8-octanediylbis[2,2-dichloro-acetamide], also called WIN 18,446), which acts by inhibiting ATRA synthesis [36], reversibly inhibits spermatogenesis in men by inducing an arrest of germ cell differentiation at the spermatogonia stage [37,38], which resembles the phenotype we describe here in the mouse. Thus it is possible that RAR/RXR heterodimers also drive SALL4A expression in human spermatogonia.

### A model for the effects of ATRA on KIT expression

A hallmark of the transition to a differentiating state in spermatogonia is the expression of KIT receptor at the surface of A_1_ spermatogonia [5]. However, it is unclear what regulatory steps control the expression of this crucial cell surface receptor. Several studies have shown that undifferentiated spermatogonia are primed to turns on KIT and initiate differentiation upon activation of an ATRA signal [2]. This ATRA signal acts indirectly on *Kit* because its mRNA is not induced by BMS961, as assessed from our microarray expression profiling (see above) and no RAR binding site is found in *Kit* [39]. From our present study, we propose that ATRA enhances the level of *Sall4a* mRNA, allowing thereby an increase of the amount of SALL4A in spermatogonia. In agreement with this proposal, SALL4A appears at PN3-PN4 in wild-type spermatogonia [31,32], coinciding with the onset of endogenous ATRA signaling [40] and differentiation of the first KIT-positive spermatogonia [41]. SALL4A in high amount could (i) sequester ZBTB16, resulting in the release of the ZBTB16-mediated repression of *Kit* expression [14]; (ii) interact with DNMT3A and/or DNMT3B [42], allowing the epigenetic shift which is instrumental to A_1_ transition to take place properly [43]; and (iii) act on yet unknown other components of the differentiation program required in spermatogonia to transition to the A_1_ state.

In agreement with our proposal, Hobbs et al. [14] reported that the total amount of SALL4 protein detected in spermatogonia is higher at the A_1_ stage (*i*.*e*., in the ZBTB16-high, KIT-positive cell population) than at the A_al_ stage (*i*.*e*., in the ZBTB16-high, KIT-negative cell population). It is however not possible to show by IHC that A_al_ spermatogonia expressing SALL4A upon ATRA signaling activation differentiate into A_1_ spermatogonia and express KIT because, contrary to what has been stated in a previous report [31], antibodies to SALL4 do not distinguish between SALL4A and SALL4B. As SALL4B is expressed in spermatogonia and in their precursors from embryonic day 17.5 onwards [31,32], these antibodies are unsuitable to detect a specific increase in the expression of the sole SALL4Aisoform.

**Fig 8:**
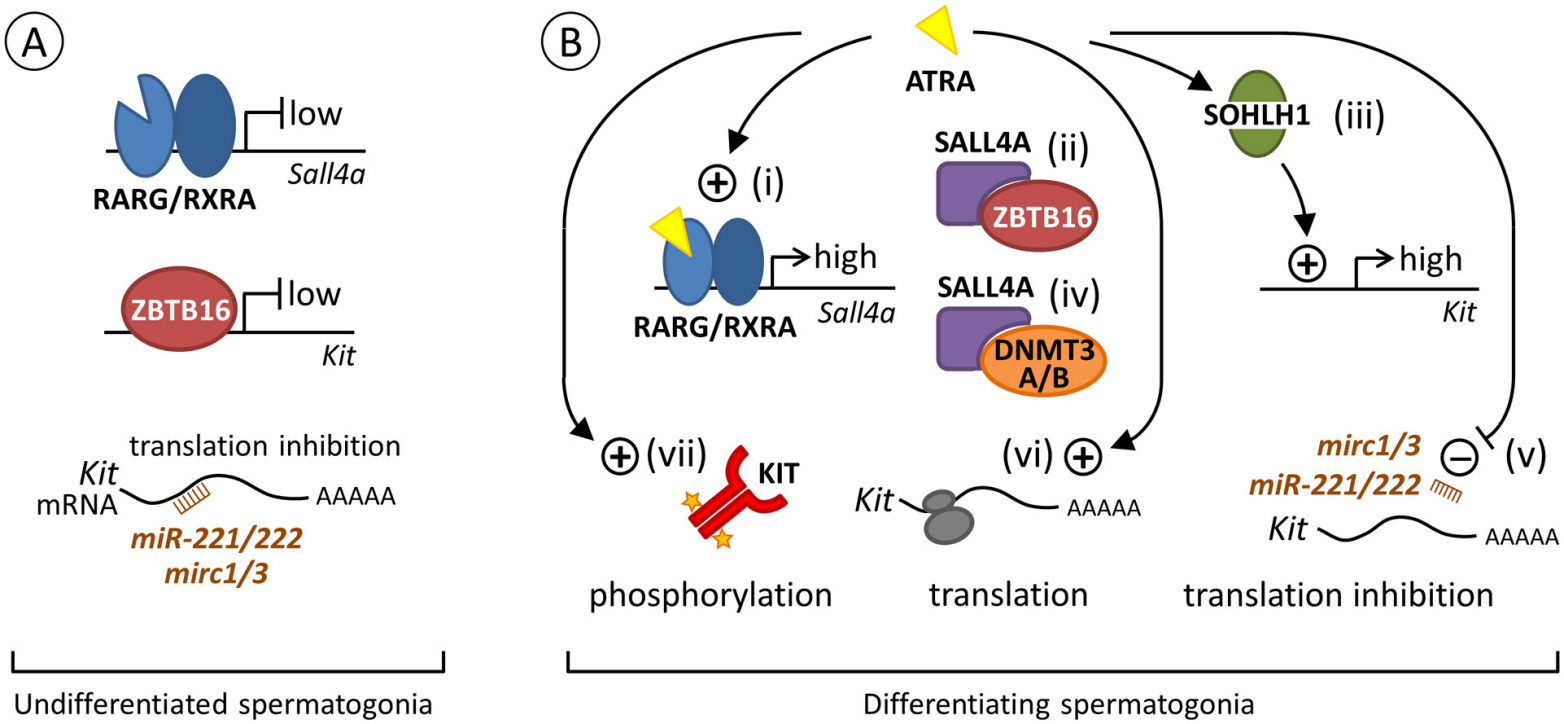
Proposed model for the regulation of *Kit* expression by ATRA during the transition from A_al_ to A_1_ spermatogonia. (A) Spermatogonia at an undifferentiated state. ATRA is not available to activate RARG/RXRA heterodimer and transcription of *Sall4* is low. Transcription of *Kit* is also low because ZBTB16 is bound to its promoter [48]. In addition, translation of *Kit* mRNA already present in cells [2,49,50] is prevented by the *Mirc1*, *Mirc3* and *miR221/222* small interfering RNAs (brown comb) [50,51]. (B) Spermatogonia at a differentiating state upon ATRA action. (i) At the A_al_-A_1_ transition, one possible way for ATRA (yellow triangle) to regulate *Kit* expression is to activate RARG/RXRA heterodimer, which increases *Sall4a* expression (high, our study). (ii) SALL4A in large amount can then sequesters ZBTB16 [14], clearing *Kit* promoter and relieving the repression of *Kit* transcription normally exerted by ZBTB16 [48]. (iii) ATRA is also proposed to increase the level of SOHLH1, which can replace ZBTB16 on regulatory regions to increase *Kit* expression (high) [52]. *Sohlh1* is however not a direct target of RARG as RAR-binding sites are not found in this gene [34] and its expression is not induced by BMS961 (our study). (iv) Alternatively, SALL4A can also interact with DNMT3A/B to facilitate the epigenetic shift required for A_1_ differentiation [42,43]. (v) In parallel, ATRA can further induce KIT protein through decreasing expression of microRNA such as *Mirc1*, *Mirc3* and *miR221/222* that prevent *Kit* mRNA translation. How ATRA regulate miRNA expression is however unknown, as RARE have not been identified in the vicinity of their promoters [34,50,51]. ATRA can also function as a rapid, non-genomic, agent by (vi) increasing the loading of *Kit* mRNA on polysomes (grey ovals) and its translation [41] and (vii) inducing phosphorylation of KIT (orange stars) and of downstream KIT-effectors, reinforcing commitment towards the A_1_ spermatogonia fate [53].

A comprehensive model summarizing the combination of transcriptional, post-transcriptional and non-genomic effects of ATRA pathways possibly controlling KIT expression and the commitment of A_al_ spermatogonia towards the A_1_ fate is proposed (Fig 8). The interest of better understanding the control of KIT expression in spermatogonia is not restricted to gametogenesis, but extends to testicular cancer. In fact, seminoma cells frequently bear somatic mutations activating KIT, or overexpress KIT or SALL4 [7,44,45]. Therefore, pharmacological modulation of mechanisms that regulate KIT expression in spermatogonia, such as antagonizing ATRA action, might have important applications for future therapeutic strategies.

## Materials and Methods

### Mice and treatments

Mice were on a mixed C57BL/6-129/Sv (50–50%) genetic background. They were housed in a licensed animal facility (agreement #A67-218-37). All experiments were approved by the local ethical committee (Com’Eth, accreditations #2012–080 and #2012–081), and were supervised by N.B.G. or M.M. who are qualified in compliance with the European Community guidelines for laboratory animal care and use (2010/63/UE). To inactivate *Rar*- or *Rxr*-coding genes in spermatogonia, mice carrying *lox*P-flanked alleles (L2) of *Rara*, *Rarb* and *Rarg* or of *Rxra*, *Rxrb* and *Rxrg* [12, and references therein] were crossed with mice bearing the *Tg(Stra8-cre)* ^*1Reb*^ transgene [16]. In F1, *Rxra* ^*L2/L2*^;*Rxrb* ^*L2/L2*^;*Rxrg* ^*L2/L2*^ females were crossed with males bearing one copy of the transgene (*Stra8* ^*tg/0*^). The resulting males (*Stra8-Cre* ^*tg/0*^;*Rxra* ^*+/L2*^;*Rxrb* ^*+/L2*^;*Rxrg* ^*+/L2*^) were backcrossed on *Rxra* ^*L2/L2*^;*Rxrb* ^*L2/L2*^;*Rxrg* ^*L2/L2*^ females to generate mutant males in F2 (*Stra8-Cre* ^*tg/0*^;*Rxra* ^*L2/L2*^;Rxrb^*L2/L2*^;*Rarg* ^*L2/L2*^), and their control littermates (*Rxra* ^*+/L2*^;*Rxrb* ^+/L2^;*Rxrg* ^*+/L2*^ and *Rxra* ^*L2/L2*^;*Rxrb* ^*L2/L2*^;*Rxrg* ^*L2/L2*^ males). The same approach was used to inactivate *Rara*, *Rarb* and *Rarg* in spermatogonia. *Aldh1a1-3* ^*ser-/-*^ mutants were generated as described previously [13]. Mice lacking RARA in Sertoli cells in a RARG-null genetic background were obtained by crossing *Rara* ^Ser–/–^[12] and *Rarg* ^–/–^ mice [9] together. BMS961 (50 mg/kg body weight, Tocris Bioscience) dissolved in dimethylsulfoxide was administered to the mice by intra peritoneal injections. BrdU (Sigma-Aldrich) was dissolved in phosphate buffered saline and injected by intra peritoneal at 50 mg/kg body weight.

### Isolation of germ cell populations by flow cytometry and organotypic cultures

Germ cell populations were purified from testes of *Aldh1a1-3* ^*ser-/-*^, *Rara;b;g* ^*Spg–/–*^ and *Rxra;b;g* ^*Spg–/–*^ mice by FACS and characterized as described previously [13,22]. Organotypic cultures of testes from *Aldh1a1-3* ^*ser-/-*^ mice were also as described previously, except that the RARG-selective agonist BMS961 at 10^–7^M (Tocris Bioscience) was used to activate RAR signaling instead of BMS753 [13].

### Histology, detection of apoptotic and proliferating cells, immunohistochemistry (IHC) and *in situ* hybridization (ISH)

For histology, testis samples were fixed in Bouin’s fluid for 16 hours and embedded in paraffin. Histological sections (5 μm-thick) were stained with hematoxylin and eosin or with periodic acid-Shiff (PAS). The percentage of affected seminiferous tubules was established on PAS-stained histological sections by counting cross-sections of tubules (n > 400 per testis). For all other methods, testes were fixed for 16 hours in 4% (wt/vol) buffered paraformaldehyde (PFA). For detection of apoptotic cells, TUNEL assays were performed using the *In Situ* Cell-Death Detection kit, Fluorescein (Roche Diagnostics). BrdU incorporation was detected by using an anti-BrdU antibody (Roche Molecular Biochemicals) and immunofluorescence labeling as described [9]. For IHC, 10 μm-thick frozen sections were incubated overnight at 4°C with rabbit anti-STRA8 (Ab49602, Abcam), rabbit anti-RARG1 (D3A4 #8965, Cell Signaling Technology), goat anti-ZBTB16 (AF2944, R&D Systems) and rabbit anti-KIT (D13A2 #3074, Cell Signaling Technology) antibodies diluted 1:200 to 1:500. Detection of bound primary antibodies was achieved by incubating the section with Cy3-conjugated goat anti-rabbit IgG (Jackson ImmunoResearch) or Alexa Fluor 488-conjugated donkey anti-goat IgG (Life Technologies). ISH using digoxigenin-labeled probes for detection of *Gfra1*, *Kit*, *Stra8* and *Zbtb16* expression was performed as described [9,10,20]. The sections were all counterstained with 0.001% (vol/vol) 4,6-diamidino-2-phenylindole dihydrochloride (DAPI) and mounted in Vectashield (Vector Laboratories). The pattern of *Cre* expression driven by *Tg(Stra8-cre)* ^*1Reb*^ [16] was assessed through testing excision in mice carrying the *Gt(ROSA)* ^*26Sortm1Sor*^ reporter transgene [46]. In these mice, *E*. *coli* beta-galactosidase is synthesized only in cells that have experienced Cre-mediated deletion of an intervening stop sequence. Analysis of beta-galactosidase activity was as described [10].

### Analysis of RNA and chromatin

Total RNA was prepared using TRIzol reagent (Life Technologies). Reverse transcription of total RNA followed by PCR amplification of cDNA was performed using QuantiTect Reverse Transcription (Qiagen) and LightCycler 480 SYBR Green I Master (Roche Diagnostics) kits, respectively. Primers were as indicated in Table 1. Triplicates of at least three samples were used in each experimental condition. The transcript levels were normalized relative to that of *Rplp0* or *Gapdh* transcripts, whose expressions are not changed by retinoid administration. Data were expressed as fold induction relative to vehicle or control conditions. To prepare chromatin, PN5 testes were fixed with 0.4% PFA (wt/vol) for 15 minutes, before being sonicated to shear DNA to an average size of 500 bp. For each reaction, 100 μg of chromatin was first incubated with 18 μg of ChIP grade anti-RAR (sc-773; Santa Cruz biotechnology), anti-RXR (sc-774; Santa Cruz biotechnology) or anti-RNA polymerase II (RNApol2; sc-9001; Santa Cruz biotechnology) antibodies and then with protein G-Sepharose. Beads were washed, and eluted DNA–protein complexes were reverse cross-linked and purified. ChIP was performed in triplicate, using distinct chromatin extracts. The recovered immuno-precipitated DNA was analyzed by triplicate qPCR and was compared with input DNA. Quantitation was determined by the enrichment of the binding site compared with a site located upstream the TSS (–11kb), and were expressed as mean fold-enrichment (n = 3). The sequences of the oligonucleotides used are indicated in Table 2. Statistical significance was assessed by Student *t* tests or by one-way ANOVA followed by the *post hoc* Newman-Keuls test for comparison by pairs.

**Table 1:** Primers used in quantitative RT-PCR.

Gene	Accession no.	Primers	Position (nt)	Size (nt)
*Gapdh*	NM_001289726.1	5’-AAGGTCATCCATGACAACTT-3’	570–657	88
		5’-GGCCATCCACAGTCTTCTGG-3’		
*Rara*	NM_001177	5’-AGCACCAGCTTCCAGTCAGT-3’	569–733	165
		5’-AGTGGTAGCCGGATGATTTG-3’		
*Rarb*	NM_011243	5’-GCTGGGTCGTCGTTTTCTAA-3’	1167–1282	135
		5’-GAAACAGGCCTTCTCAGTGC-3’		
*Rarg*	NM_011244.4	5’-CTCGGGTCTATAAGCCATGC-3’	746–805	60
		5’-CCCCATAGTGGTAGCCAGAA-3’		
*Rplp0*	NM_007475	5’-ACCCTGAAGTGCTCGACATC-3’	720–908	208
		5’-AGGAAGGCCTTGACCTTTTC-3’		
*Rxra*	NM_001290481.1	5’-GATATCAAGCCGCCACTAGG-3’	384–534	151
		5’-TTGCAGCCCTCACAACTGTA-3’		
*Rxrb*	NM_001205	5’-GGGCTGCAAGGGTTTCTTCA-3’	186–348	163
		5’-CTCCTGAACCGCCTCCCTTT-3’		
*Rxrg*	NM_009107	5’-TGTGGTCAACAGTGTCAGCA-3’	6–190	185
		5’-AGAAGCCTTTGCAACCTTCA-3’		
*Sall4a*	NM_175303.4	5’-AGTGTCACCTGCCAATAGCC-3’	2560–2726	167
		5’-TGCCAGGCACTTCAACTTT-3’		
*Sall4b*	NM_201395.3	5’-CTCGACCAGTCCAAGAAAGG-3’	1232–1394	163
		5’-TGCCAGGCACTTCAACTTT-3’		
*Zbtb16*	NM_001033324.2	5’-AACGGTTCCTGGACAGTTTG-3’	1563–1734	172
		5’-CCACACAGCAGACAGAAGA-3’		

Gene names, accession numbers, forward (upper line) and reverse (lower line) primers, their positions in the sequences and sizes of the amplified fragments are indicated. nt: nucleotide.

**Table 2:** Primers used for ChIP and EMSA assays.

Gene	Primer	Assay
*IR1*	5’-ATGAGGTAGAAGGTCACTGAACTTTGATAAACTCG-3’	EMSA
	5’-CGAGTTTATCAAAGTTCAGTGACCTTCTACCTCAT-3’	
*mutated IR1*	5’-ATGAGGTAGAAAGGCGCTGAACTTTGATAAACTCG-3’	EMSA
	5’-CGAGTTTATCAAAGTTCAGCGCCTTTCTACCTCAT-3’	
*DR0*	5’-GCAGGCGGGCTGAGGGTTAACCCTTTTGTT-3’	EMSA
	5’-AACAAAAGGGTTAACCCTCAGCCCGCCTGC-3’	
*DR1*	5’-CATCGCAGGACCAGGGGAGGGGTCAGGGCC-3’	EMSA
	5’-GGCCCTGACCCCTCCCCTGGTCCTGCGATG-3’	
*Rarb DR5*	5’-CGGGTAGGGTTCACCGAAAGTTCACTCGCA-3’	EMSA
	5’-TGCGAGTGAACTTTCGGTGAACCCTACCCG-3’	
*Sall4*	5’-AGCAATGACCTTCCAGTTGC-3’	ChIP
	5’-TGGGATCCTACTTTTCCCAAA-3’	
*Sall4 –11kb*	5’-TTGATCGGACAGCTTTTGTG-3’	ChIP
	5’-GGGACTGGAGGGAGAAAAAG-3’	
*Stra8 –3kb*	5’-GGCAGCAGGCCACCAATAAA-3’	ChIP
	5’-TAGGCTTGGTTCCCCGTGTG-3’	
*Stra8 DR2*	5’-AGGTCATCTTGCTCCTTCCA-3’	ChIP
	5’-ATCACAGCCCTGTCACTGC-3’	
*Stra8 DR4*	5’-GTGAGGTAGATCCCGGATTG-3’	ChIP
	5’-GACCTGAGGTGAGCTGCTTC-3’	

Gene, forward (upper line) and reverse (lower line) primers and their use are indicated. IR0, inverted repeats separated by 0 nucleotide; DR0, DR1, DR2, DR4 and DR5, direct repeats separated by 0, 1, 2, 4 and 5 nucleotides, respectively.

### Electrophoretic mobility shift assays

They were performed as described previously [47]. Briefly, the oligonucleotides were annealed and labeled with [γ-32P]ATP (Amersham Bioscience). For competition assays, unlabeled oligonucleotides were added in the incubation mixture in 1- to 1000-fold molar excess. The sequences of the oligonucleotides used are described in Table 2.

### Western blotting

Protein extracts were prepared in 50 mM Tris-HCl (pH7.5) buffer containing 150 mM NaCl, 0.5% (wt/vol) sodium deoxycholate, 1% (vol/vol) NP40, 0.2% (vol/vol) sodium dodecyl sulfate (SDS), and protease inhibitor mixture (Roche diagnostics). They were resolved by 4–16% (wt/vol) gradient SDS polyacrylamide gel electrophoresis (Expedeon) and transferred to nitrocellulose membranes (Protran) using standard protocols. The membranes were incubated with anti-SALL4 antibodies (Ab29112; Abcam) diluted 1:500 and IgG were detected using goat anti-mouse coupled to horseradish peroxidase as secondary antibodies (diluted 1:5000) followed by chemiluminescence according to the manufacturer’s protocol (GE Healthcare). The blots were subsequently incubated 2 times for 10 minutes at room temperature in 0.2M glycine pH 2.2 containing 0.1% (wt/vol) SDS and 0.1% (wt/vol) Tween 20, and were further probed with anti-actin antibodies (sc-58673; Santa Cruz biotechnology) diluted 1:500 to verify for equivalent loading in all the lanes.

## Supporting Information

S1 Fig The *Tg(Stra8-cre)* ^*1Reb*^ transgene allows gene ablation in spermatogonia from post-natal day 3 (PN3) onward. The pattern of Cre-mediated gene excision was assessed through testing DNA excision in mice also carrying the *Gt(ROSA)* ^*26Sortm1Sor*^ reporter transgene. In these mice, *E*. *coli* beta-galactosidase is synthesized only in cells that have experienced Cre-mediated deletion of an intervening stop sequence (Soriano, 1999). (A-C,E,F) X-Gal staining and (D) STRA8 IHC on testis sections in mice bearing both *Tg(Stra8-cre)* ^*1Reb*^ and *Gt(ROSA)* ^*26Sortm1Sor*^ transgenes. At PN3 (*i*.*e*., shortly after the onset of spermatogenesis), beta-galactosidase activity (red signal) is already initiated in about 50% of the spermatogonia and in all seminiferous cord (A,B), whereas spermatogonia expressing endogenous STRA8 are exceptional. At PN5, beta-galactosidase-positive spermatogonia become widespread (C), and staining for STRA8 on consecutive sections (D) shows that its activity is initiated within a subset of spermatogonia that outnumbers those expressing the endogenous *Stra8*. At PN20, beta-galactosidase activity is observed in all spermatocytes and spermatids (E,F). These data indicate that excision of the reporter transgene occurs in undifferentiated spermatogonia as early as PN3 and is complete in all germ cells from PN5 onwards, in accordance with previous reports [14,16]. The *Tg(Stra8-cre)* ^*1Reb*^ thus appears to be a suitable tool to study the role of RXR and RAR in spermatogonia. Note that (i) both the cytosolic and the juxta-nuclear, punctate (green arrowheads), beta-galactosidase activities indicate Cre-mediated excision of *LacZ*; (ii) in *A*, *B* and *F*, the staining resulting from beta-galactosidase activity was converted to a red false color, and the DAPI nuclear stain was converted to a bright-field image and then to a blue false color using Photoshop software; (iii) a processing artifact of consecutive sections has induced a distortion making the distances between the seminiferous cords wider in panel D than in panel C; and (iv) X-Gal-positive cells are never detected in testes of age-matched *Gt(ROSA)* ^*26Sortm1Sor*^ mice that do not harbor the *Tg(Stra8-cre)* ^*1Reb*^ transgene. The box in A indicates the region displayed at a higher magnification in B. Legend: C, seminiferous cords; G spermatogonia; PR and P, preleptotene and pachytene spermatocytes, respectively; R, round spermatids. Scale bar: 80 μm (*A* and *C-F*) and 25 μm (*B*).
(TIF)

S2 Fig Ablation of all RAR in spermatogonia induces age-related testis degeneration. (A-B) Section from 12 week-old control and *Rara;b;g* ^*Spg–/–*^ testes. (C-H) Germ cell associations in the seminiferous epithelium of *Rara;b;g* ^*Spg–/–*^ mutants. Normal gem cell associations at epithelial stage VII (C) coexist with abnormal associations lacking: pachytene spermatocytes (D,H), preleptotene spermatocytes (E,G) and round spermatids (F,H). (I,J) Section from 12 month-old control and *Rara;b;g* ^*Spg–/–*^ testes: seminiferous tubules containing only spermatogonia and Sertoli cells represent the end-stage of degeneration in the mutant testes (J). PR and P, preleptotene and pachytene spermatocytes, respectively; St7 and St16, step 7 and 16 spermatids, respectively; T2, tubules sections lacking generation(s) of germ cells around their entire circumference; T3, tubules sections with complete disorganization of the germ cell layer; T4, tubules sections containing only spermatogonia and Sertoli cells. Germ cell populations present in a given tubule cross-section are highlighted by colored bars: red, preleptotene spermatocytes; green, pachytene spermatocytes; blue, step 7 (round) spermatids; purple, step 16 (elongated, mature) spermatids. Roman numerals indicate the stages of the seminiferous epithelium cycle. Periodic acid-Schiff (A-H) and hematoxylin and eosin (I-J) stains. Scale bar, 80 μm (A-B and I-J) and 30 μm (C-H).
(TIF)

S3 Fig Ablation of *Rar* and *Rxr* genes in spermatogonia with *Tg(Stra8-cre)* ^*1Reb*^ transgene is efficient from PN3 onward. (A) Relative expression of *Rarg* and *Rxra* mRNA quantified by RT-qPCR in whole testes from control (white bars), *Rara;b;g* ^*Spg−/−*^ (grey bars) and *Rxra;b;g* ^*Spg−/−*^ (black bars) mice at PN5 (upper panel) and PN60 (lower panel). Error bars represent s.e.m. (n = 5); * *p* < 0.05. (B) PCR analysis of genomic DNA extracted from FACS-purified germ cells in heterozygote control mice (left panel) and *Rara;b;g* ^*Spg−/−*^ mutant mice (right panel) at PN60. This experiment proves efficient excision of the *Rara*, *Rarb* and *Rarg* alleles in all germ cells populations isolated from the mutant testes, as assessed by the absence of *Rara* L2 alleles and the trace amounts of *Rarb* or *Rarg* L2 alleles, which might be attributed to a low, contaminating, number of somatic cells. SG, PR, Z/P, P and RS, purified germ cell populations containing spermatogonia, preleptotene/leptotene spermatocytes, zygotene/early pachytene spermatocytes, late pachytene/diplotene spermatocytes and round spermatids, respectively. L2 and L–, conditional (*lox*P-flanked) and null (excised) alleles, respectively. CTL– and CTL+, negative (no DNA added) and positive (L2/L2 or L–/L–DNA added) control PCR reactions, respectively. (C-F) Immunohistochemical detection of spermatogonia expressing RARG (red nuclear signal) in control and *Rara;b;g* ^*Spg–/–*^ testes at PN5 and PN60. ZBTB16 expression (green nuclear signal) identifies spermatogonia. Spermatogonia nuclei co-expressing RARG and ZBTB16 appear in yellow. VII, stage VII of the seminiferous epithelium cycle; SG, spermatogonia. PR, preleptotene spermatocytes; asterisks indicate non-specific fluorescence in Leydig cells. Scale bars: 55 μm (C and D) and 40 μm (E and F).
(TIF)

S4 Fig Both RAR and RXR are bound to *Stra8* promoter in mouse testis. Schematic representation of *Stra8* locus and analysis by qPCR of DNA recovered from PN5 wild-type testis chromatin immunoprecipitated using antibodies directed against RNApol2, all RAR isotypes (RAR) or all RXR isotypes (RXR) at the *Stra8* locus. The untranslated exons and the two transcription start sites (TSS1 and TSS2) are depicted by open boxes and broken arrows, respectively. The locations of primers used for qPCR are indicated at −3 kb and in *Stra8*. Mean fold enrichment of three experiments at DR4 (grey bars) and DR2 (black bars) binding sites is relative to the amount of DNA recovered at −3 kb (set at 1, white bars). Error bars represent s.e.m (n = 5); * *p* < 0.05.
(TIF)

S5 Fig RARG/RXRA heterodimers bind to the DR1, DR0 and IR1 motifs of *Sall4* RAR-binding region. EMSA showing that RARG/RXRA heterodimers (Het) bound to the DR5 of *Rarb* (lane 3) are competed both when unlabeled DR5 (lane 5) or increasing amounts of DR1 (lanes 6–8), DR0 (lanes 9–11) and IR1 (lanes 12–14) are added to reaction. ^32^P-DR5 probe indicates unbound DNA.
(TIF)
