# Supplementary figures and images for: Retinoic Acid Receptors Control Spermatogonia Cell-Fate and Induce Expression of the SALL4A Transcription Factor
Source: PLoS Genet. 2015 Oct 1;11(10):e1005501. doi: 10.1371/journal.pgen.1005501 (PMC4591280; doi:10.1371/journal.pgen.1005501)

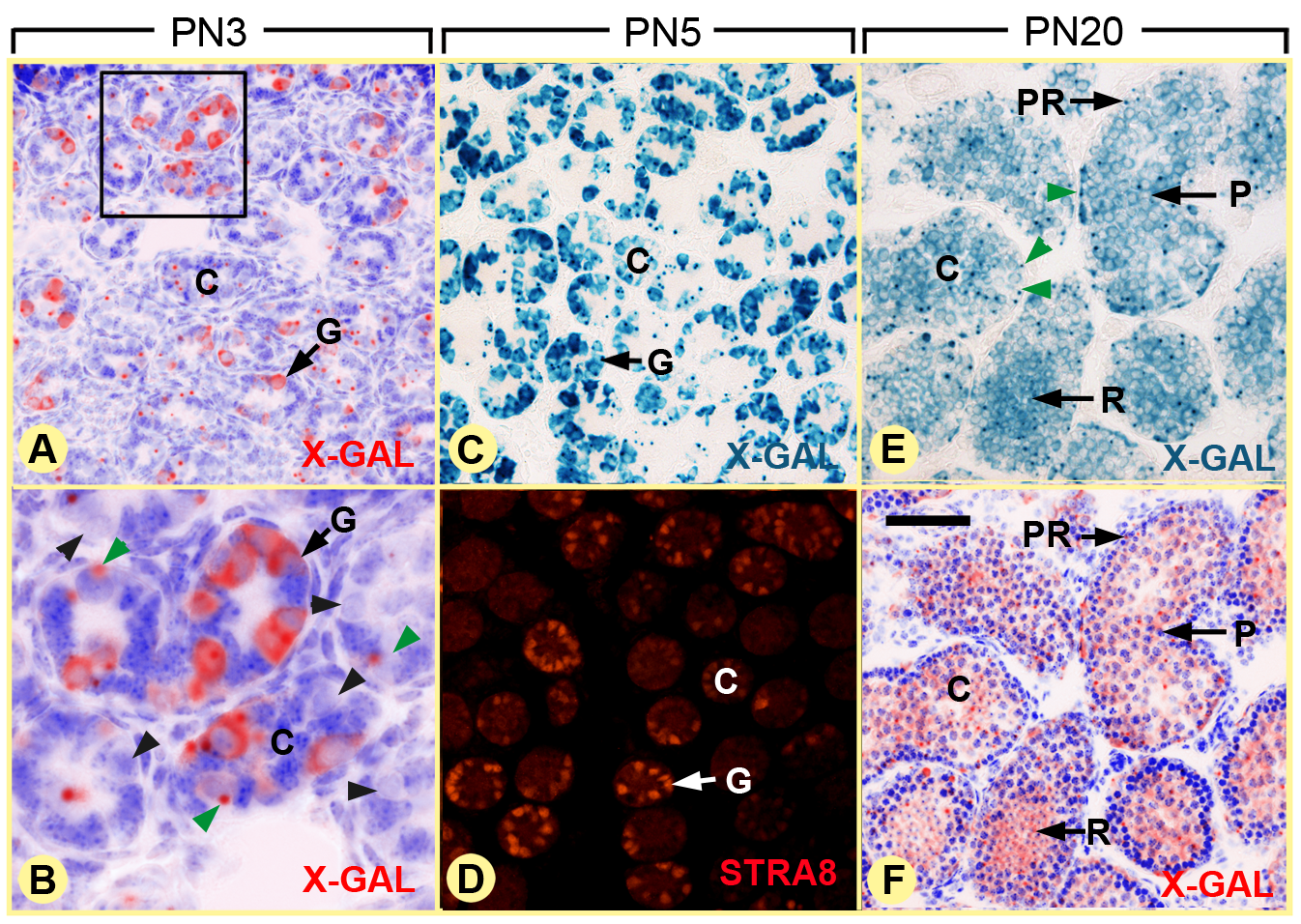

Supplement: S1 Fig — The pattern of Cre-mediated gene excision was assessed through testing DNA excision in mice also carrying the Gt(ROSA) 26Sortm1Sor reporter transgene. In these mice, E. coli beta-galactosidase is synthesized only in cells that have experienced Cre-mediated deletion of an intervening stop sequence (Soriano, 1999). (A-C,E,F) X-Gal staining and (D) STRA8 IHC on testis sections in mice bearing both Tg(Stra8-cre) 1Reb and Gt(ROSA) 26Sortm1Sor transgenes. At PN3 (i.e., shortly after the onset of spermatogenesis), beta-galactosidase activity (red signal) is already initiated in about 50% of the spermatogonia and in all seminiferous cord (A,B), whereas spermatogonia expressing endogenous STRA8 are exceptional. At PN5, beta-galactosidase-positive spermatogonia become widespread (C), and staining for STRA8 on consecutive sections (D) shows that its activity is initiated within a subset of spermatogonia that outnumbers those expressing the endogenous Stra8. At PN20, beta-galactosidase activity is observed in all spermatocytes and spermatids (E,F). These data indicate that excision of the reporter transgene occurs in undifferentiated spermatogonia as early as PN3 and is complete in all germ cells from PN5 onwards, in accordance with previous reports [14,16]. The Tg(Stra8-cre) 1Reb thus appears to be a suitable tool to study the role of RXR and RAR in spermatogonia. Note that (i) both the cytosolic and the juxta-nuclear, punctate (green arrowheads), beta-galactosidase activities indicate Cre-mediated excision of LacZ; (ii) in A, B and F, the staining resulting from beta-galactosidase activity was converted to a red false color, and the DAPI nuclear stain was converted to a bright-field image and then to a blue false color using Photoshop software; (iii) a processing artifact of consecutive sections has induced a distortion making the distances between the seminiferous cords wider in panel D than in panel C; and (iv) X-Gal-positive cells are never detected in testes of age-matche [file pgen.1005501.s001.tif]

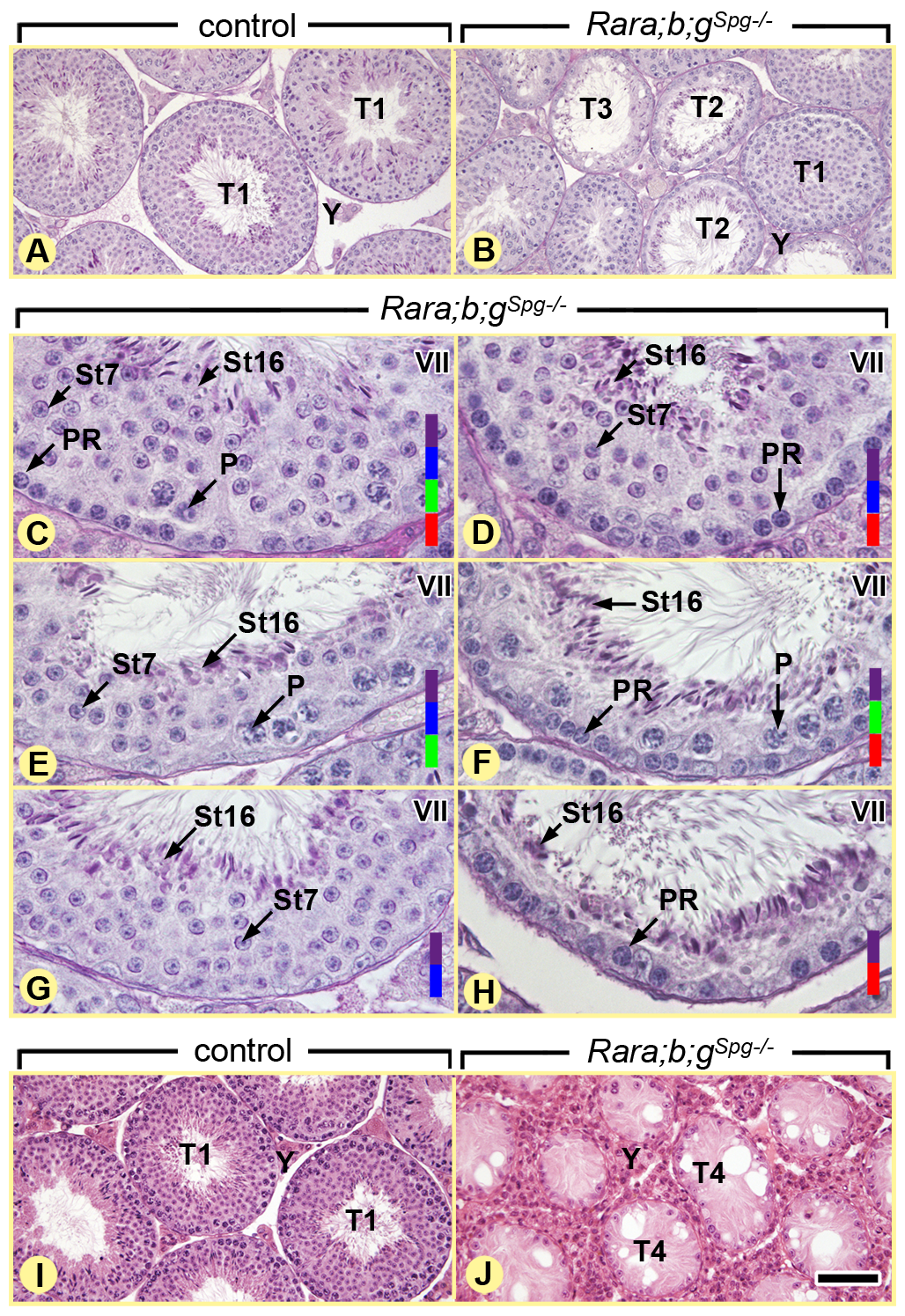

Supplement: S2 Fig — (A-B) Section from 12 week-old control and Rara;b;g Spg–/– testes. (C-H) Germ cell associations in the seminiferous epithelium of Rara;b;g Spg–/– mutants. Normal gem cell associations at epithelial stage VII (C) coexist with abnormal associations lacking: pachytene spermatocytes (D,H), preleptotene spermatocytes (E,G) and round spermatids (F,H). (I,J) Section from 12 month-old control and Rara;b;g Spg–/– testes: seminiferous tubules containing only spermatogonia and Sertoli cells represent the end-stage of degeneration in the mutant testes (J). PR and P, preleptotene and pachytene spermatocytes, respectively; St7 and St16, step 7 and 16 spermatids, respectively; T2, tubules sections lacking generation(s) of germ cells around their entire circumference; T3, tubules sections with complete disorganization of the germ cell layer; T4, tubules sections containing only spermatogonia and Sertoli cells. Germ cell populations present in a given tubule cross-section are highlighted by colored bars: red, preleptotene spermatocytes; green, pachytene spermatocytes; blue, step 7 (round) spermatids; purple, step 16 (elongated, mature) spermatids. Roman numerals indicate the stages of the seminiferous epithelium cycle. Periodic acid-Schiff (A-H) and hematoxylin and eosin (I-J) stains. Scale bar, 80 μm (A-B and I-J) and 30 μm (C-H). (TIF) [file pgen.1005501.s002.tif]

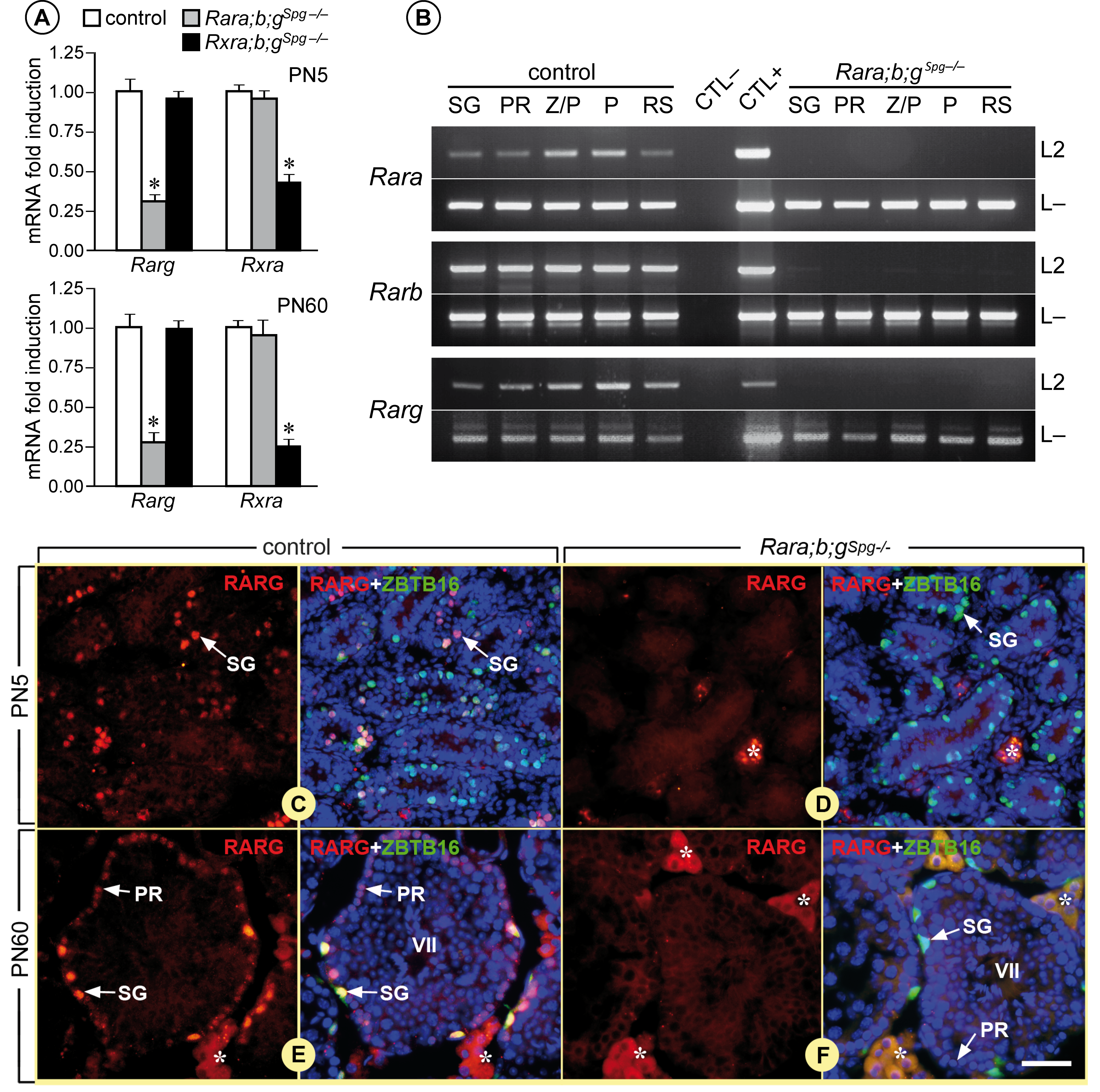

Supplement: S3 Fig — (A) Relative expression of Rarg and Rxra mRNA quantified by RT-qPCR in whole testes from control (white bars), Rara;b;g Spg−/− (grey bars) and Rxra;b;g Spg−/− (black bars) mice at PN5 (upper panel) and PN60 (lower panel). Error bars represent s.e.m. (n = 5); * p < 0.05. (B) PCR analysis of genomic DNA extracted from FACS-purified germ cells in heterozygote control mice (left panel) and Rara;b;g Spg−/− mutant mice (right panel) at PN60. This experiment proves efficient excision of the Rara, Rarb and Rarg alleles in all germ cells populations isolated from the mutant testes, as assessed by the absence of Rara L2 alleles and the trace amounts of Rarb or Rarg L2 alleles, which might be attributed to a low, contaminating, number of somatic cells. SG, PR, Z/P, P and RS, purified germ cell populations containing spermatogonia, preleptotene/leptotene spermatocytes, zygotene/early pachytene spermatocytes, late pachytene/diplotene spermatocytes and round spermatids, respectively. L2 and L–, conditional (loxP-flanked) and null (excised) alleles, respectively. CTL– and CTL+, negative (no DNA added) and positive (L2/L2 or L–/L–DNA added) control PCR reactions, respectively. (C-F) Immunohistochemical detection of spermatogonia expressing RARG (red nuclear signal) in control and Rara;b;g Spg–/– testes at PN5 and PN60. ZBTB16 expression (green nuclear signal) identifies spermatogonia. Spermatogonia nuclei co-expressing RARG and ZBTB16 appear in yellow. VII, stage VII of the seminiferous epithelium cycle; SG, spermatogonia. PR, preleptotene spermatocytes; asterisks indicate non-specific fluorescence in Leydig cells. Scale bars: 55 μm (C and D) and 40 μm (E and F). (TIF) [file pgen.1005501.s003.tif]

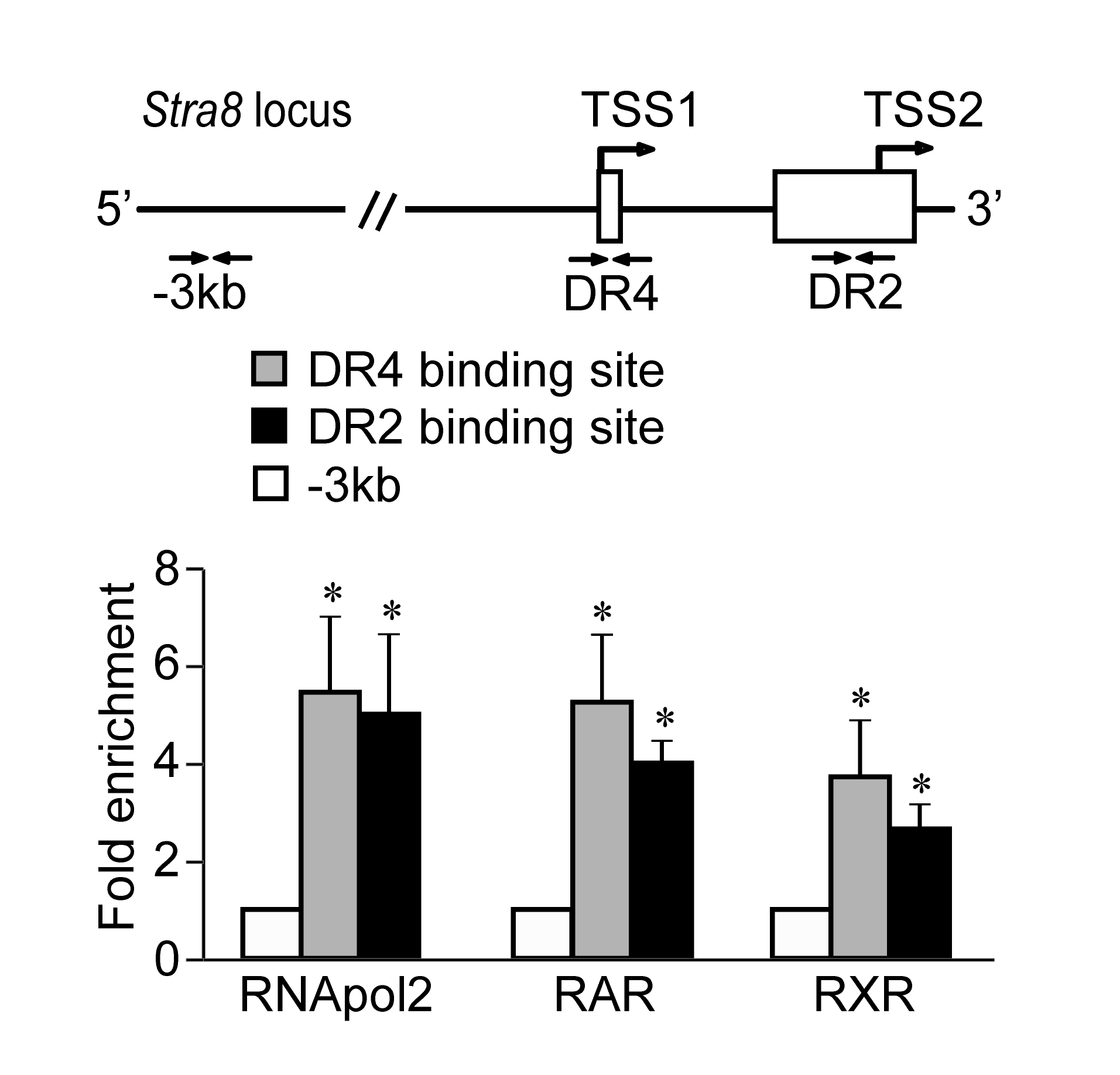

Supplement: S4 Fig — Schematic representation of Stra8 locus and analysis by qPCR of DNA recovered from PN5 wild-type testis chromatin immunoprecipitated using antibodies directed against RNApol2, all RAR isotypes (RAR) or all RXR isotypes (RXR) at the Stra8 locus. The untranslated exons and the two transcription start sites (TSS1 and TSS2) are depicted by open boxes and broken arrows, respectively. The locations of primers used for qPCR are indicated at −3 kb and in Stra8. Mean fold enrichment of three experiments at DR4 (grey bars) and DR2 (black bars) binding sites is relative to the amount of DNA recovered at −3 kb (set at 1, white bars). Error bars represent s.e.m (n = 5); * p < 0.05. (TIF) [file pgen.1005501.s004.tif]

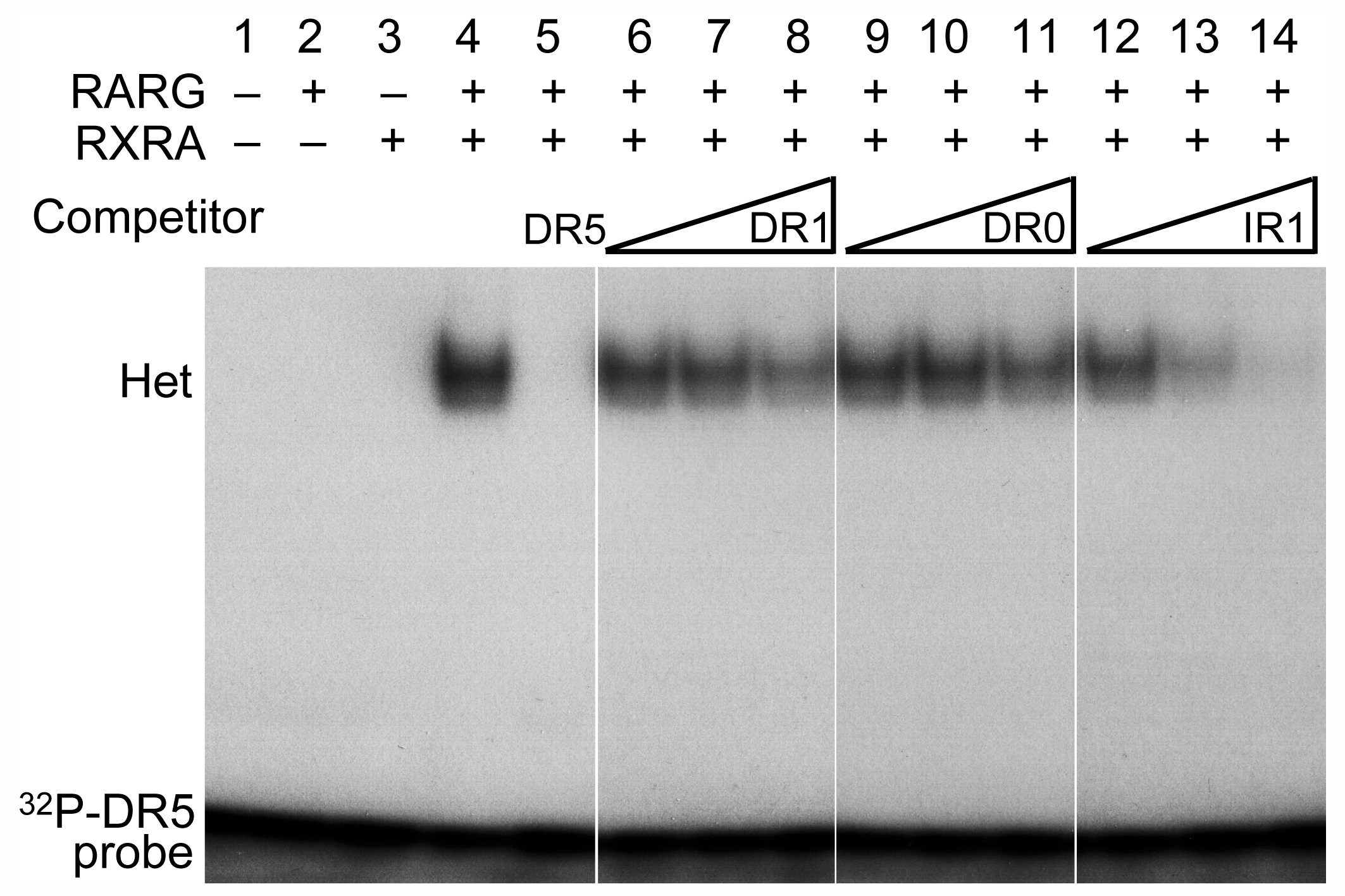

Supplement: S5 Fig — EMSA showing that RARG/RXRA heterodimers (Het) bound to the DR5 of Rarb (lane 3) are competed both when unlabeled DR5 (lane 5) or increasing amounts of DR1 (lanes 6–8), DR0 (lanes 9–11) and IR1 (lanes 12–14) are added to reaction. 32P-DR5 probe indicates unbound DNA. (TIF) [file pgen.1005501.s005.tif]
